# Topology Outweighs Stiffness: Self-Reinforced Cell Mechanotransduction via Multiaxial Curvature Engineering of Ultrasoft Hydrogels

**DOI:** 10.1021/acsnano.5c19367

**Published:** 2026-02-24

**Authors:** Yong Hou, Xinhao Hu, Cheng Qian, Wenyan Xie, Linjie Ma, Luyao Zhang, Xiaomei Han, Youhua Tan, Yuan Lin, Chao Fang, Zhiqin Chu

**Affiliations:** † Department of Electrical and Electronic Engineering, 25809 The University of Hong Kong, Pok Fu Lam 00000, Hong Kong, China; ‡ School of Science, 529484 Harbin Institute of Technology, Shenzhen 518055, Guangdong, China; § Department of Biomedical Engineering, 494312 Hong Kong Polytechnic University, Kowloon, Hong Kong 999077, China; ∥ Department of Mechanical Engineering, The University of Hong Kong, Pok Fu Lam, Hong Kong 00000, China; ⊥ School of Biomedical Sciences, The University of Hong Kong, Pok Fu Lam, Hong Kong 00000, China; # School of Biomedical Engineering, The University of Hong Kong, Pok Fu Lam, Hong Kong 00000, China

**Keywords:** multiaxial curvatures, ultrasoft substrate, mechanosensing, curvature engineering, tissue engineering

## Abstract

Geometric curvature critically regulates cellular behavior in soft tissue microenvironments, yet its role in mechanotransduction is underexplored due to stiffness-centric paradigms and challenges in creating stable curvatures on ultrasoft materials. We developed a solvent-induced buckling strategy to engineer multiaxial curvatures on ultrasoft hydrogels (500–750 Pa), recapitulating the anisotropic topologies of natural tissues such as cerebral gyri and breast lobules. Human mesenchymal stem cells on these surfaces exhibit robust focal adhesion maturation, cytoskeletal reorganization, nuclear mechanosensing (e.g., elevated Lamin A/C), and enhanced osteogenesis—phenotypes typically seen on rigid substrates but markedly attenuated on flat ultrasoft controls. This curvature-dominated mechanosensing persists in 3D injectable microgels, decoupling topological cues from the substrate stiffness. Mechanistic studies and energy minimization modeling reveal that curvature segregates stress fiber functions: basal fibers align circumferentially in high-curvature regions to enhance Rho-mediated contractility and focal adhesions, while apical fibers orient radially in low-curvature zones to minimize the bending energy. These findings establish topology as a primary driver of cellular tension and fate, providing fundamental insights into designing biomaterials and biointerfaces for soft tissue repair and regenerative medicine.

## Graphical abstract

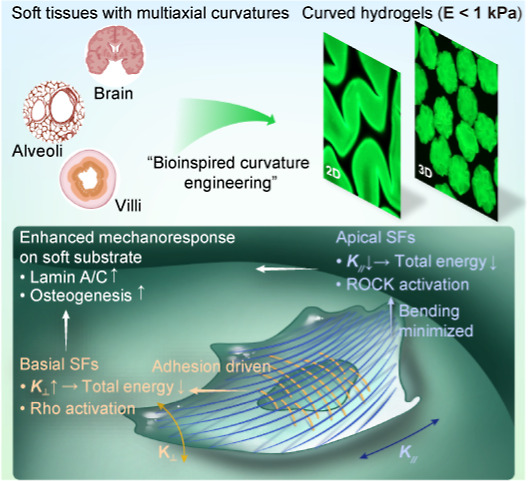

In living systems, geometric curvature serves as a fundamental mechanical cue to regulate cell behavior across scales—from the undulating folds of cerebral gyri that guide neural stem cell polarity to the branched vasculature where endothelial cells align under hemodynamic shear stress.^1–3^ Importantly, these natural architectures are not merely structural motifs but are evolution-optimized designs that spatially orchestrate cellular functions. For instance, the hyperboloidal curvature of trabecular bone enhances load-bearing efficiency while providing niches for osteoprogenitor cell adhesion and mineralization.^4^ Similarly, intestinal villi leverage concave–convex curvature gradients to compartmentalize stem cell populations and maintain Wnt/BMP signaling.^5,6^

Despite the evident connection between curvature and cellular activities, engineered curvature models have long been confined to rigid or semirigid materials, such as PDMS grooves,^7–9^ polymers,^10^ or glass/silica,^11,12^ with stiffness ranging from hundreds of kPa to GPa. Early studies showed that convex surfaces on stiff substrates promote osteogenesis by enhancing cytoskeletal tension and nuclear deformation, while concave surfaces facilitate the formation of epithelial junctions.^1,13,14^ However, these systems cannot disentangle the individual roles of curvature and substrate stiffness and do not replicate the ultralow stiffness microenvironments encountered by cells in soft tissues (e.g., brain 0.1–1 kPa, villi ∼1 kPa, Figure 1a–c). This limitation may stem from the long-held stiffness-centric dogma of mechanobiology, which prioritizes substrate rigidity as the primary driver of cell fate and function.^15^ However, recent studies have shown that for ultrasoft matrices (<1 kPa), topological factors, such as surface pattern and curvature, could override or dominate the default low-tension state induced by low stiffness.^16–18^ For instance, Shukla et al. reported that patterned grooves on soft hydrogels promote human mesenchymal stem cells (hMSCs) alignment and osteogenic differentiation.^19^ Interestingly, Cui et al. found that in ultrasoft three-dimensional environments, cells adhere to matrix fibers through integrin α_v_β_5_-mediated curved adhesions, which are driven by matrix curvature rather than stiffness.^20^ These findings indicate that when mechanical resistance from the surrounding microenvironment is minimal, cells may prioritize geometric cues over stiffness in adjusting their activities.

**Figure 1:**
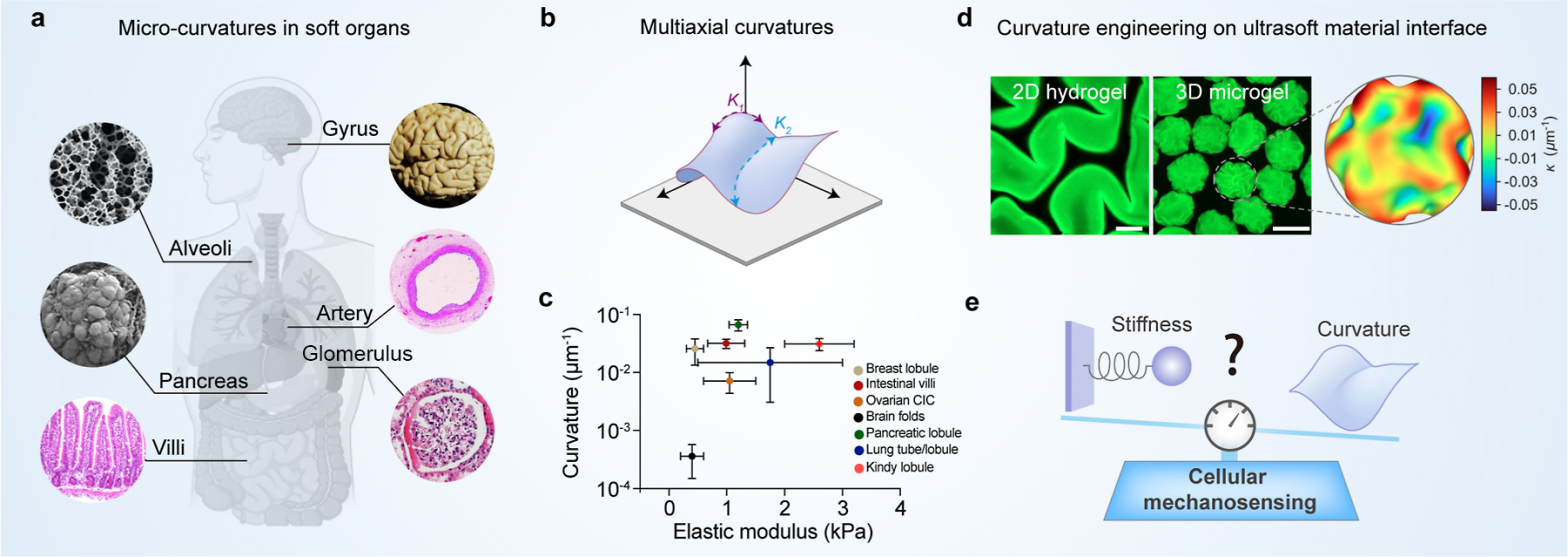
Multiaxial microcurvatures in soft tissues and their engineering on ultrasoft materials. (a) Schematic illustrating multiaxial microcurvatures in various soft human tissues (e.g., brain gyri, intestinal villi, lung alveoli, kidney glomeruli), highlighting their ubiquitous presence across different length scales in the human body.^25–30^ Alveoli: adapted, with permission of the © 2019 Elsevier Ltd.;^31^ The images of gyrus,^29^ villi,^30^ and artery^28^ are adapted with licensed under CC BY 4.0. Pancreas: adapted with permission of ©Taylor & Francis Group;^26^ glomerulus: adapted with permission of © 2010 Gu and Herrera, publisher and licensee Dove Medical Press Ltd.^26^ The (b) Conceptual framework of multiaxial curvature, where curvature (*K*) is defined as the inverse of the radius of a circle tangential to the surface at the corresponding point.^1^ (c) Statistical plot comparing stiffness (kPa) and curvature scales of various soft tissues, derived from previous reported data.^32–48^ (d) Fluorescence microscopy images showcasing engineered multiaxial microcurvatures on ultrasoft 2D hydrogels and 3D microgels, replicating biological curvature features. An enlarged view of the 3D microgel surface, paired with a color map, illustrates the spatial variation and complexity of curvature profiles. Scale bar: 200 μm. (e) Conceptual diagram framing the study’s central question: how multiaxial curvature amplifies cellular stress perception and mechanosensing on ultrasoft interfaces, potentially superseding the role of substrate stiffness.

Another unresolved issue is that existing curvature paradigms predominantly focus on single-axis geometries (e.g., cylindrical) or isotropic multiaxial surfaces (e.g., spherical, with uniform curvature across axes), which fail to capture the complex shape and anisotropic nature of in vivo microenvironments. Practically, due to swelling/deswelling induced distortion, introducing stable curvatures on soft hydrogels is known to be notoriously difficult. Furthermore, commonly used surface feature-creating techniques such as high-resolution 3D printing often struggle with ultralow-modulus materials. These difficulties hinder both mechanistic exploration of curvature-sensing pathways and translational applications in regenerative medicine, such as in fibrosis, where tissue stiffening alters the matrix curvature,^21^ or cancer, where the tumor cells’ (e.g., pancreatic cancer) proliferation and metastasis rely on their adaptation to the complex-shaped microenvironments.^3^

To address these gaps, we developed a solvent-induced buckling strategy to engineer multiaxial curvatures on the surface of ultralow stiffness hydrogels (0.5–4.3 kPa). Similar to the self-organization of cerebral cortical folds and vascular networks, this method generates stable microcurvatures without compromising the softness of the material (Figure 1d,e). We found that hMSCs on these ultrasoft curved surfaces (<1 kPa) exhibit robust focal adhesion (FA) maturation, cytoskeletal rearrangement, and nuclear mechanosensing activation—phenotypes reminiscent of those typically observed on flat stiff substrates, yet absent on flat ultrasoft controls. Crucially, this “curvature-dominated mechanosensing” persists in 3D injectable ultrasoft microgels, demonstrating the decoupling of topological guidance from mechanical stiffness. Through inhibition assays, transcriptomic profiling, and theoretical modeling based on energy minimization principles, we further elucidated the key pathways by which multiaxial curvature is transduced into biochemical signals.

By isolating curvature’s effects, we reveal a new mechanotransduction paradigm where topology, not stiffness, dictates cellular tension and fate. This could redefine how we model tissue development or design biomaterials for soft tissue repair,^22,23^ offering alternative therapeutic strategies for diseases where curvature plays a pivotal role.^24^

## Results and Discussion

### Curvature-Engineering on Ultrasoft Hydrogels

To mimic multiaxial curvatures exhibited by many biological tissues, we developed a solvent-induced curvature engineering (SICE) strategy by leveraging the swelling/deswelling kinetics of hydrogels. As shown in Figure 2a, cross-linked gelatin methacryloyl (GelMA) hydrogel films were photopolymerized on methacrylated glass slides. After immersing in ethanol (a poor solvent for GelMA hydrogel), the diffusion of ethanol molecules induces rapid dehydration of the hydrogel network, leading to a heterogeneous deswelling process.^49–51^ This deswelling generates higher compressive stresses at the gel surface due to the presence of more solvent molecules than that in the bulk.^50,52^ Upon rehydration in water, the differential swelling between the ethanol-treated surface (with higher cross-link density due to localized dehydration) and the hydrated bulk created a stress gradient. This gradient triggered mechanical instabilities, forming stable wrinkled patterns akin to those observed in constrained swelling hydrogels.^52^ The extent of surface deformation was found to increase with the stress disparity between the surface and the bulk, modulated by factors such as solvent quality, cross-link density, and solvent dielectric constant.

**Figure 2:**
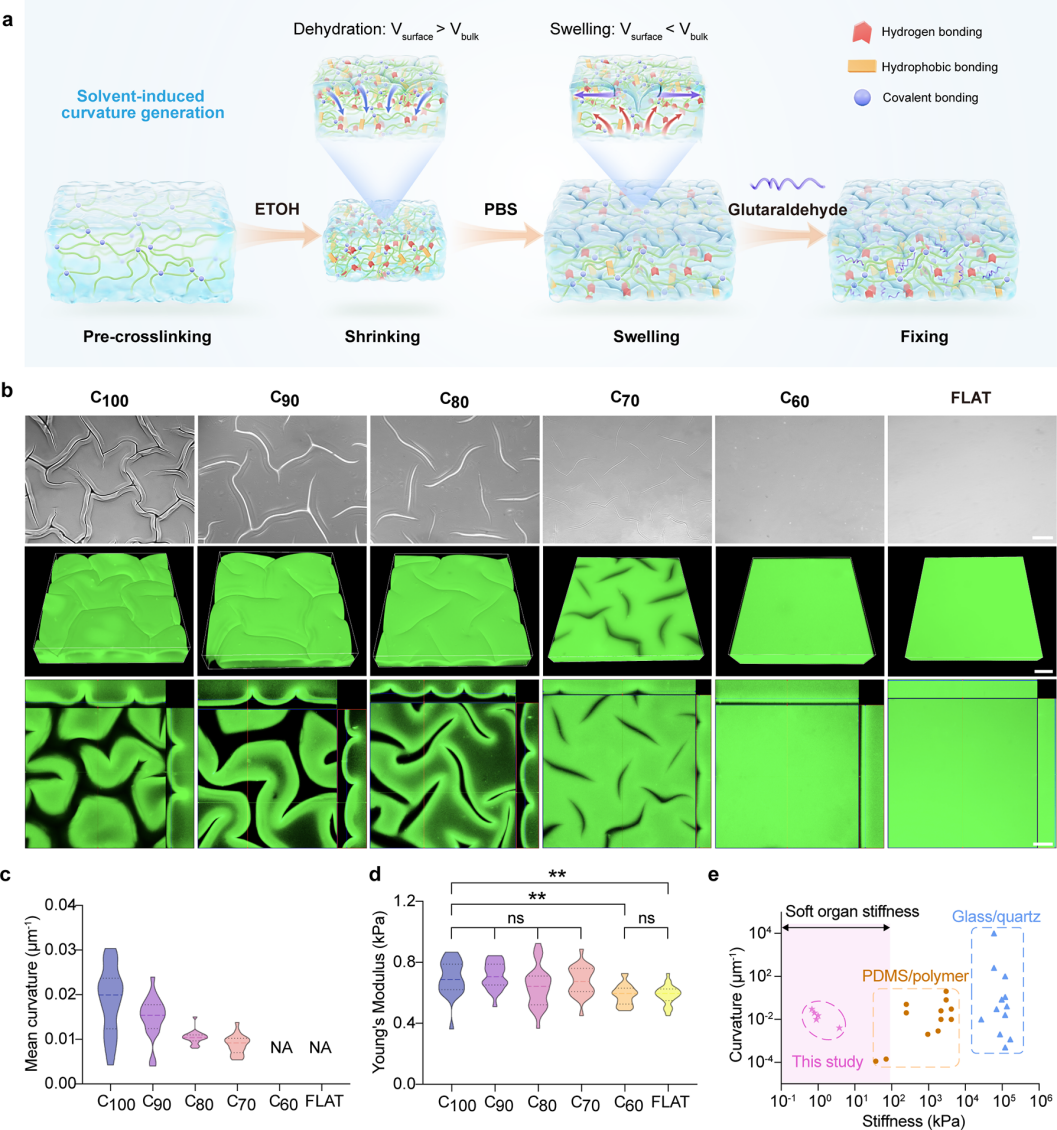
Fabrication and characterization of multiaxial curvature on gelatin hydrogels via SICE. (a) Schematic illustrating the SICE method for generating multiaxial curvatures on hydrogel substrates. (b) Bright-field and 3D-reconstructed fluorescence microscopy images showing the surface morphology of hydrogels treated with different ethanol–water ratios (100%, 90%, 80%, 70%, 60%, and 0% ethanol by volume). Scale bar: 200 μm. (c) Measurement of mean curvature of hydrogel substrates under different ethanol–water treatment conditions (*N* = 5, three technical replicates). (d) Quantification of Young’s modulus of hydrogel substrates with varying curvature levels (*N* = 5, three technical replicates). (e) Plot comparing the curvature and Young’s modulus ranges achieved in this study (ultrasoft hydrogels) with those of prior curvature models (e.g., glass, PDMS molds).^54–66^ Data represent mean ± s.d. *P < 0.05, **P < 0.01, ***P < 0.001, one-way ANOVA. ns, not significant.

Specifically, the ultrasoft GelMA hydrogel (∼ 690 Pa, 4.5 wt %) was chosen as a model system in the present study to investigate curvature formation at biointerfaces. The morphology of the wrinkled surface of GelMA hydrogels was examined across ethanol–water mixtures ranging from 100% to 0% ethanol (Figure 2b). As the ethanol content decreased, the depth and width of groove-like structures diminished gradually (e.g., depth from 155 to 30.8 μm, width from 85.9 to 26.4 μm, Figure S1). The mean curvature shifted from 0.019 ± 0.007 μm^–1^ in pure ethanol to 0.009 ± 0.002 μm^–1^ in 70% ethanol (Figure 2c). This trend is consistent with the Flory–Huggins theory, which predicts that poor solvents (like ethanol, with a high interaction parameter χ) reduce polymer swelling and promote network dehydration.^49^ Higher ethanol concentrations intensify surface dehydration, leading to localized polymer densification. Upon rehydration in water, differential swelling between the dehydrated surface and the hydrated bulk induces compressive stress, resulting in buckling and stable wrinkle formation. Atomic force microscope (AFM) results confirmed a slight stiffness increase (∼100 Pa) in curved regions after SICE treatment (FLAT vs C_100_), indicating the “physical crosslinking” induced by the dehydration process (Figure 2d).

Physically, the magnitude of the resulting surface curvature is closely tied to compressive strains arising from differential swelling and deswelling, which are strongly influenced by the hydrogel’s cross-link density.^52,53^ To verify this, we evaluated the dehydration response of GelMA hydrogels with varying cross-link densities (4.5%–20% w/v) in pure ethanol. Hydrogels with lower cross-link density exhibited significantly larger curvatures post-SICE treatment; for instance, the mean curvature in 4.5% GelMA was approximately two times of that in 10% GelMA (e.g., 0.019 ± 0.006 μm^–1^ vs 0.008 ± 0.002 μm^–1^, Figure S2). This is because loosely cross-linked networks permit greater solvent uptake and swelling, leading to higher deswelling strain and thus larger compressive stress and curvature upon buckling—as predicted by elastic deformation principles.

Furthermore, the solvent dielectric constant (ε) emerged as another critical factor governing curvature generation.^49^ As a weak polyelectrolyte, gelatin’s swelling behavior should be modulated by solvent dielectric constant. A low dielectric constant solvent could suppress ionic osmotic pressure and therefore enhance deswelling and the hydrogel.^51^ Indeed, by using ethanol (ε = 24.6), isopropanol (ε = 18.3), acetone (ε = 20.7), methanol (ε = 32.6), acetonitrile (ε = 36.6), DMF (ε = 36.7), and DMSO (ε = 46.7) in our experiments, we found that solvents with high dielectric constant (e.g., acetonitrile, DMF, DMSO) produced little surface wrinkling on the gel, while low- ε solvents (e.g., methanol, acetone, isopropanol) induced pronounced curvatures (Figure S3).

In summary, the SICE strategy enables precise and stable curvature engineering on ultrasoft hydrogels, achieving a tunable mean curvature ranging from 0.007 to 0.035 μm^–1^ on substrates with stiffnesses spanning from 0.3 to 4.3 kPa (Figure S2). This stiffness range closely mimics that of human soft tissues, such as the brain (0.1–1 kPa), liver (1–5 kPa), and adipose tissue (0.5–10 kPa) (Figure 2e), in direct contrast to conventional curvature models that rely on materials such as polydimethylsiloxane (PDMS,4–100 kPa to MPa), glass, or quartz (GPa range). Moreover, we confirmed that the SICE principle is readily applicable to other widely used natural and synthetic hydrogel systems (alginate, matrigel, and polyacrylamide), yielding similar curvatures (Figure S4), which underscores its potential broad applicability across material platforms. Importantly, the surface curvatures introduced via our approach remain stable for at least 1 week at 37 °C, ensuring suitability for long-term cell culture experiments (Figure S5).

### Curvatures on an Ultrasoft Substrate Enhance hMSCs Spreading, Nuclear Mechanics, and Differentiation

To investigate how multiaxial curvatures regulate hMSCs behavior in a tissue-mimicking microenvironment, we utilized GelMA hydrogels treated with ethanol/water mixtures at 100% (C_100_), 70% (C_70_), and 0% (FLAT) to create high-, moderate-, and low-curvature interfaces, respectively. These ultrasoft hydrogels form irregular wrinkled structures, generating multiaxial curvature interfaces that modulate the cell adhesion and morphology. Figure 3 illustrates hMSCs spreading on these substrates after 24 h of culture, with 3D reconstructions of image stacks visualizing F-actin cytoskeleton organization and cell morphology.

**Figure 3:**
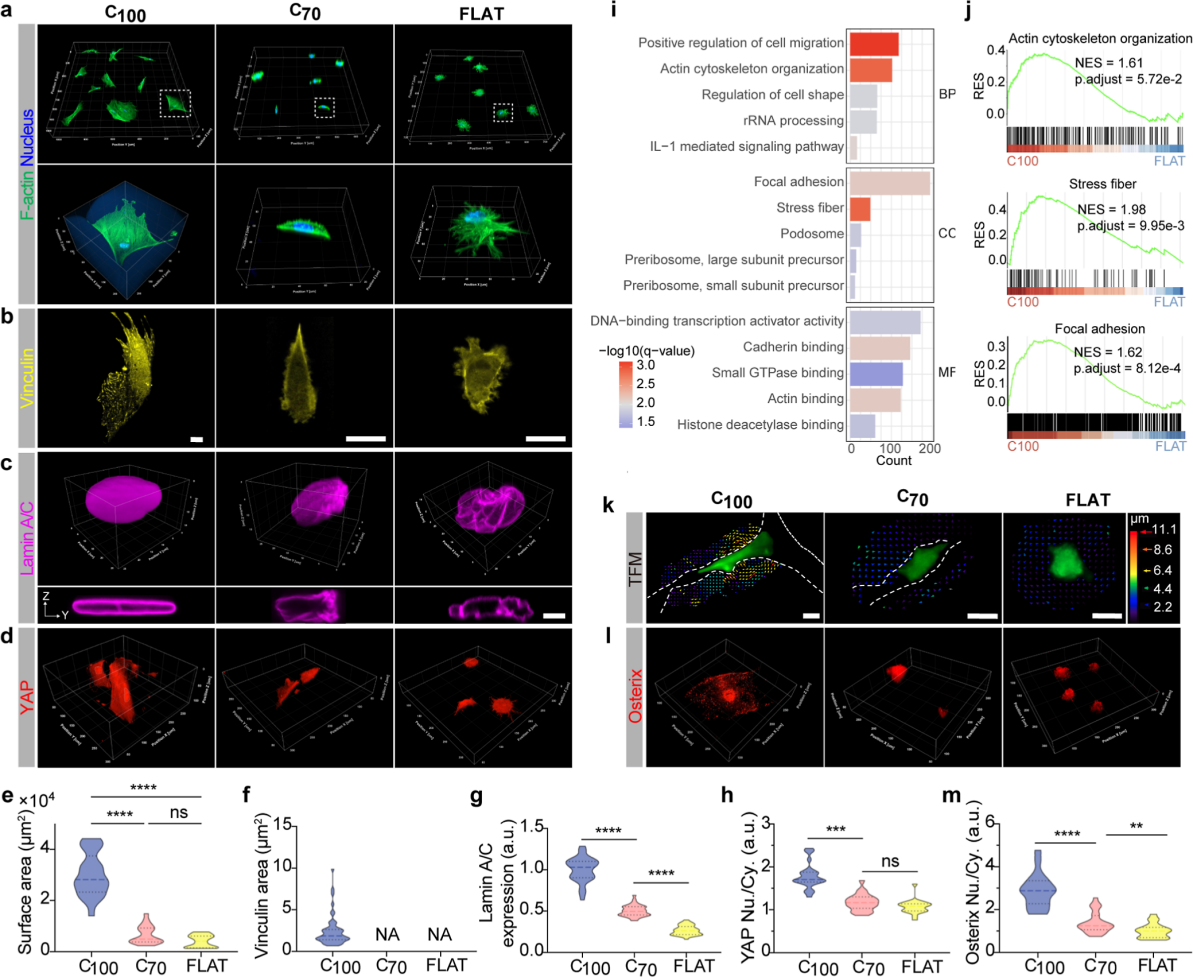
Multiaxial curvature on ultrasoft substrate regulates hMSCs spreading, differentiation, and mechanosensing. (a,b) Immunofluorescence staining of F-actin (green), nuclei (blue), and vinculin (yellow) of hMSCs on 2D hydrogels with different curvatures. Scale bar: 10 μm. (c) Immunofluorescence images of Lamin A/C (magenta) in hMSCs on soft hydrogels with varied curvatures after 12 h of culture in growth medium. Insets provide Y-Z cross-sectional views of nuclear morphology, illustrating curvature-induced changes in nuclear architecture. Scale bar: 50 μm. (d) Representative fluorescence images of hMSCs stained with anti-YAP (red) on soft hydrogels with varied curvatures after 12 h in the growth medium. (e–h) Quantification of the cell surface area, vinculin area, Lamin A/C expression, and Yes-associated protein (YAP) nuclear/cytoplasmic ratio of hMSCs on a soft hydrogel with varied curvatures. (*N* = 25–40, three technical replicates). (i) Gene Ontology (GO) analysis of differentially expressed genes (DEGs) in hMSCs cultured on 100% curvature (C_100_) versus flat (FLAT) hydrogels. (j) Gene Set Enrichment Analysis (GSEA), focusing on pathways related to actin cytoskeleton organization, stress fiber (SF), and FA in response to curvature. (k) Traction force measurements illustrating substrate mechanical remodeling by hMSCs on ultrasoft hydrogels with different curvatures. Scale bar: 20 μm. (l) Representative fluorescence images of hMSCs stained for osterix (red) on soft hydrogels with varying curvatures after 3 days in osteogenic medium. (m) Quantification of the osterix nuclear-to-cytoplasmic ratio in hMSCs on soft hydrogels with varying curvatures (*N* = 30–40, three technical replicates). Data represent mean ± s.d. **P < 0.01, ***P < 0.001, one-way ANOVA.

Our results demonstrate that hMSCs adapt their morphology to substrate curvature, with the spreading area increasing proportionally with the curvature (Figure 3a,b). On high-curvature C_100_ substrates, cells predominantly adhered to one side of groove-like structures, displaying a robust, dense F-actin cytoskeleton and enhanced FA formation compared to C_70_ and flat substrates (Figure 3a,b,e,f). Notably, cell shape and cytoskeletal alignment followed the substrate’s curvature, exhibiting bending in both the *X*–*Y* plane (along the groove) and the X–Z circumferential direction. On moderate-curvature C_70_ substrates, cell adhesion was reduced, with the surface area measuring 23% of that on C_100_, yet a “contact guidance” effect persisted, as cells polarized along the groove direction without significant X–Z bending (Figure 3a,b). In contrast, on flat substrates, hMSCs exhibited limited mechanosensing due to insufficient mechanical feedback from the ultrasoft substrate (586 ± 64 Pa), resulting in spherical morphology and minimal FA maturation (Figure 3a,b,e,f). To isolate curvature effects from substrate stiffness, we compared cell spreading on substrates with stiffness ranging from 546 to 911 Pa, observing no significant differences (Figure S6). Furthermore, to rule out the potential contributory effect of the slight stiffness increase induced by SICE treatment, we fabricated flat hydrogels with a Young’s modulus precisely matched to the C_100_ condition (∼700 Pa). Cells on these stiffness-matched flat substrates exhibited significantly inferior spreading area, Lamin A/C expression, and YAP nuclear localization compared to those on the curved C_100_ substrates (Figure S7). Together, these results confirm that the robust mechanosensing on C_100_ substrates is driven dominantly by multiaxial curvature, not by the concomitant minor change in substrate stiffness.

To examine nuclear mechanoresponse, we stained Lamin A/C, a nuclear cytoskeleton protein and a marker of nuclear tension. Cells with high nuclear tension exhibited elevated Lamin A/C expression and a smooth nuclear envelope, whereas those with low tension showed reduced Lamin A/C and a wrinkled nuclear morphology.^16^ On C_100_ substrates, hMSCs displayed significantly higher Lamin A/C expression and larger, smoother nuclei compared to C_70_ and flat substrates (Figures 3c,g and S8), consistent with increased cellular contractility. Treatment with Blebbistatin and Latrunculin B, inhibitors of myosin and F-actin polymerization, respectively, markedly reduced Lamin A/C expression and induced wrinkled nuclear morphology (Figure S9), indicating that curvature-induced actomyosin contractility regulates nuclear mechanics. YAP1, a mechanosensitive transcriptional regulator, was assessed to further evaluate cellular mechanical responses.^67^ YAP1 nuclear accumulation increased with substrate curvature, showing 1.7-fold and 1.1-fold higher levels on C_100_ and C_70_ substrates, respectively, compared to flat surfaces (Figure 3d,h), confirming that higher curvature induces greater cellular tension.

To elucidate underlying mechanisms, bulk RNA-seq analysis of hMSCs on C_100_ and flat substrates revealed DEGs enriched in GO pathways related to mechanotransduction, including actin cytoskeleton organization, SF assembly, FA, actin binding, and small GTPase signaling (Figure 3i,j). GSEA indicated significantly higher enrichment of these pathways in C_100_-cultured cells compared to flat substrates, suggesting upregulation of actin-cytoskeleton-related genes to sustain elevated cellular mechanics.

Traction force microscopy (TFM) confirmed that hMSCs on high-curvature C_100_ substrates exhibited the highest traction forces, followed by C_70_ and flat substrates (Figure 3k). Given that high cellular forces promote hMSCs osteogenesis, we cultured cells in a differentiation medium on substrates with varying curvatures. As expected, C_100_ substrates, with the highest curvature, significantly enhanced osteogenesis, as evidenced by increased expression of osterix, an early osteogenic marker (Figure 3l,m). Collectively, these data demonstrate that multiaxial curvatures on ultrasoft substrates reorganize actin and focal adhesions, enhancing hMSCs mechanosensing, nuclear mechanics, and osteogenic differentiation.

### SFs and Focal Adhesions Align in Multidirectional Patterns in Response to the Multiaxial Curvatures on Soft Hydrogels

Our SICE method generates ultrasoft hydrogel substrates with intricate multiaxial curvatures through random wrinkle formation. This complex wrinkled topography comprises regions of both positive (convex, *K* > 0) and negative (concave, *K* < 0) curvatures (Figures 1d, 2b and 4a). However, our experimental observations revealed that hMSCs predominantly adhered to and spread along the sidewalls of the wrinkle ridges, which exhibit a positive curvature (convex-like features), rather than residing in the troughs with a negative curvature (Figures 3a, 4b and S7a). This preferential adhesion dictated the primary cell–substrate interactions and the resultant mechanophenotypes. Therefore, to elucidate the dominant curvature-sensing mechanism, this study focuses on the effects of positive multiaxial curvatures.

**Figure 4:**
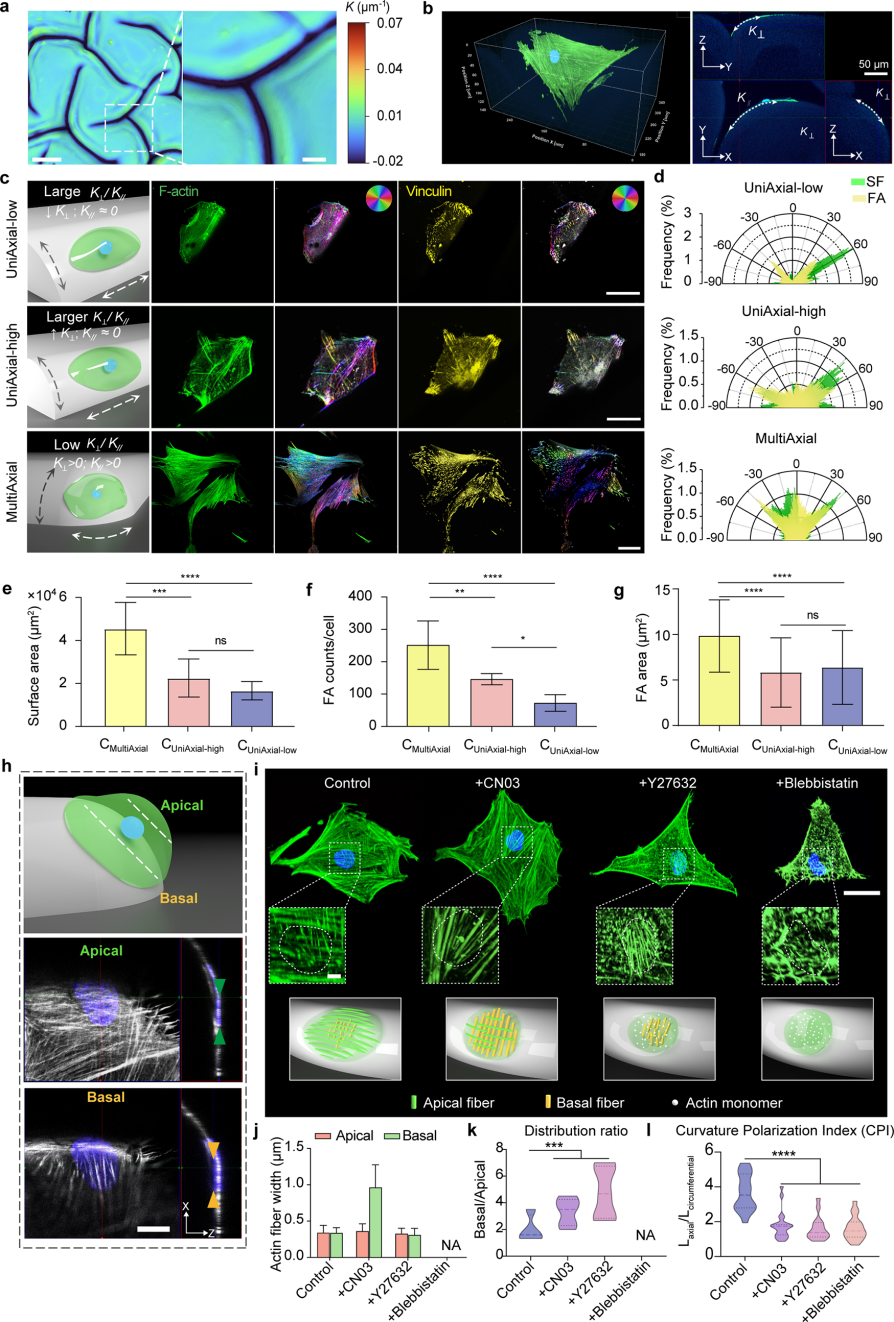
Multidirectional alignment of SFs and focal adhesions in response to multiaxial curvatures on soft hydrogels. (a) Color map illustrating the variation and complexity of curvature features on hydrogel surface post-SICE treatment. The scale bars indicate 200 μm (left) and 50 μm (right). (b) 3D confocal microscopy images of F-actin (green) in hMSCs, illustrating deformation of the cell body and actin cytoskeleton by multiaxial surface curvatures. *K*_∥_ represents the axial curvature, and the *K*_⊥_ indicates the circumferential curvature features. (c) Immunofluorescence images of F-actin (green) and vinculin (yellow) in hMSCs on UniAxial-low, UniAxial-high, and MultiAxial hydrogels, with orientation maps. Scale bar: 100 μm. (d) Polar diagram depicting the orientation distribution of SFs (green) and FAs (yellow). (e–g) Quantification of the cell surface area (*N* = 10–15, three technical replicates), FA density (*N* = 10–15, three technical replicates) and area (*N* = 40–60, three technical replicates) responding to different curvatures. (h) Schematic (upper) and immunofluorescence images (lower) of apical (above nucleus) and basal (below nucleus) SFs in hMSCs on multiaxial curvatures. Scale bar: 10 μm. (i) Immunofluorescence images of SFs (green) and nuclei (blue) in hMSCs treated with CN03 (10 μg/mL), Y27632 (10 μM), or blebbistatin (20 μM), with insets highlighting apical and basal actin distribution. Lower panel: schematic of apical (red) and basal (green) actin alignment under drug treatments. The scale bars indicate 10 μm (main), 2 μm (inset). (j,k) Measurements of apical and basal actin width and population. At least 200 SFs in 6 cells were analyzed in each condition. (l) Curvature polarization indices (CPI, defined as L_axial_/L_circumferential_) of drug-treated hMSCs (*N* = 15–20, three technical replicates). Data represent mean ± s.d. **P < 0.01, ***P < 0.001, one-way ANOVA.

To systematically analyze how cells perceive these random yet physically definable topographies, we categorized the local curvature states in cell-adhesive regions based on two principal directions: (i) quasi uniaxial curvatures (*K*_∥_ ≈ 0, *K*_⊥_ > 0), resembling groove-like patterns with primarily out-of-plane curvatures, and (ii) multiaxial curvatures (*K*_∥_ > 0, *K*_⊥_ > 0), exhibiting curvature in both groove-parallel and groove-perpendicular directions. Statistical analysis reveals a significantly higher areal proportion of multiaxial features on C_100_ compared to C_70_ substrates (Figure S10). Within uniaxial curvatures, we further categorized structures based on the magnitude of *K*_⊥_ into low uniaxial (UniAxial-low) and high uniaxial (UniAxial-high) groups to investigate the dose-dependent effect of circumferential curvature (Figure 4b).

On quasi uniaxial curvatures (*K*_∥_ ≈ 0, *K*_⊥_ > 0), hMSCs displayed significant vertical polarization of their cytoskeleton and focal adhesions. Most SFs and FAs aligned along the groove direction (with minimal curvature), with a smaller fraction oriented circumferentially (perpendicular to the groove along *K*_⊥_). As *K*_⊥_ increased from 0.011 μm^–1^ to 0.018 μm^–1^, the proportion of circumferentially aligned SFs and FAs increased by 1.7-fold and 1.35-fold, respectively (Figure 4c,d). This trend highlights a direct relationship between curvature magnitude and directional alignment of the cytoskeleton and FAs, consistent with findings on rigid cylindrical substrates.^60,68^ Notably, this alignment persisted in our ultrasoft system despite minimal mechanical resistance, emphasizing curvature as an independent mechanotransductive cue. In contrast, multiaxial curvature structures (*K*_∥_ > 0, *K*_⊥_ > 0) elicited a more complex response, with hMSCs spreading area increasing by 85.6%–153.9% compared to quasi uniaxial curvatures (Figure 4c,e). The polarization of SFs showed a wider distribution, no longer limited to orthogonal directions, reflecting the influence of multiaxial curvature (Figure 4c, low panel). Cells on these surfaces expressed numerous FAs, both peripherally and internally, with FA density (42.1%) and area (39.4%) higher than those on quasi uniaxial curvatures (UniAxial-low), respectively (Figure 4f,g). These findings indicate that multiaxial curvatures enhance cell adhesion and mechanosensing by offering multiple axes for force transmission.

To further investigate the multiaxial-curvature-induced SFs reorganization, we analyzed confocal image stacks, which revealed two distinct SF subpopulations relative to the nucleus. Apical SFs formed a perinuclear actin cap aligned axially (along the direction of the minimal curvature) above the nucleus, while basal SFs aligned circumferentially (perpendicular to the groove, along the direction of the high curvature) beneath it (Figure 4h). Previous studies on substrates with well-defined, engineered curvatures (e.g., cylindrical grooves or saddle points) and high stiffness (kPa to GPa range) have established fundamental principles of cellular curvature sensing by regulating cell actin arrangement.^4,14,68^ These works suggest that apical SFs act as topological sensors, bridging concave gaps to transmit nuclear deformation, while basal SFs align with maximal curvature due to contractility, contradicting bending energy minimization.^60^ To explore these principles in ultrasoft regime, we modulated actomyosin contractility via the Rho/ROCK pathway, a key regulator of SF formation and contractility.^69^ Rho activation with CN03, which selectively inhibits GTPase activity without affecting Rac or Cdc42,^68,70^ increased basal SF width 300% and shifted the basal-to-apical SF ratio from 35.3% to 67.9% (Figure 4i–k). ROCK inhibition with Y-27632 ablated apical SFs, leaving thin, circumferentially aligned basal SFs (Figure 4i–k), indicating that the basal SFs are more stable and resistant to Rho/ROCK perturbations compared with apical SFs. Myosin inhibition with Blebbistatin abolished actin fiber formation, reducing cell spreading by 50% compared with controls (Figure 4i–k). Similarly, the actin polymerization inhibition with 0.2 μM Latrunculin B severely impaired cell spreading and cytoskeleton development, leaving cells spherical and unable to spread (Figures S11 and S12).

To quantify curvature perception, we measured the axial (L_axial_) and circumferential (L_cir_) projection lengths of cells, defining their ratio as the Curvature Polarization Index (CPI). Rho activation with CN03 enhanced basal actin intensity and proportion, reducing CPI by 50.1%, while Y-27632 treatment suppressed apical actin, increasing basal actin proportion and reducing CPI by 51.8% compared to controls (Figure 4l). Consistent with the pivotal role of Rho/ROCK signaling, we found that the expression of MYPT1, a direct downstream target of ROCK, was significantly elevated in hMSCs cultured on C_100_ substrates compared to flat controls (Figure S13a,b). In parallel, transcriptomic analysis revealed significant upregulation of a set of key genes within this pathway (*RHOA*, *RHOB*, *ROCK1*, *ROCK2*, etc.) and its downstream effector *PPP1R12A* (which encodes MYPT1) under the high curvature (Figure S13c). These results confirm that the Rho/ROCK pathway is critical for curvature-induced cytoskeletal reorganization, particularly in aligning basal SFs with high-curvature directions.

### Mechanical Model Reveals Multiaxial Curvature Enhances Cell Mechanosensing through the Interplay between SFs Bending and Contractility-Induced Adhesion Strengthening

To understand how substrate curvatures on ultrasoft substrates regulate cellular mechanosensing, we developed a mechanical model that captures the nuclear configurations and SFs orientations (Supporting Information). Specifically, SFs are idealized as elastic Euler–Bernoulli beams with active contractile force, *F*_*a*_, anchored at both ends to the curved substrate via FAs (Figure 5a). The total energy of the basal SFs is thus given by

1 $$U_{total}^{b}=U_{bending}^{b}+U_{active}^{b}+U_{FA}^{b}$$

**Figure 5:**
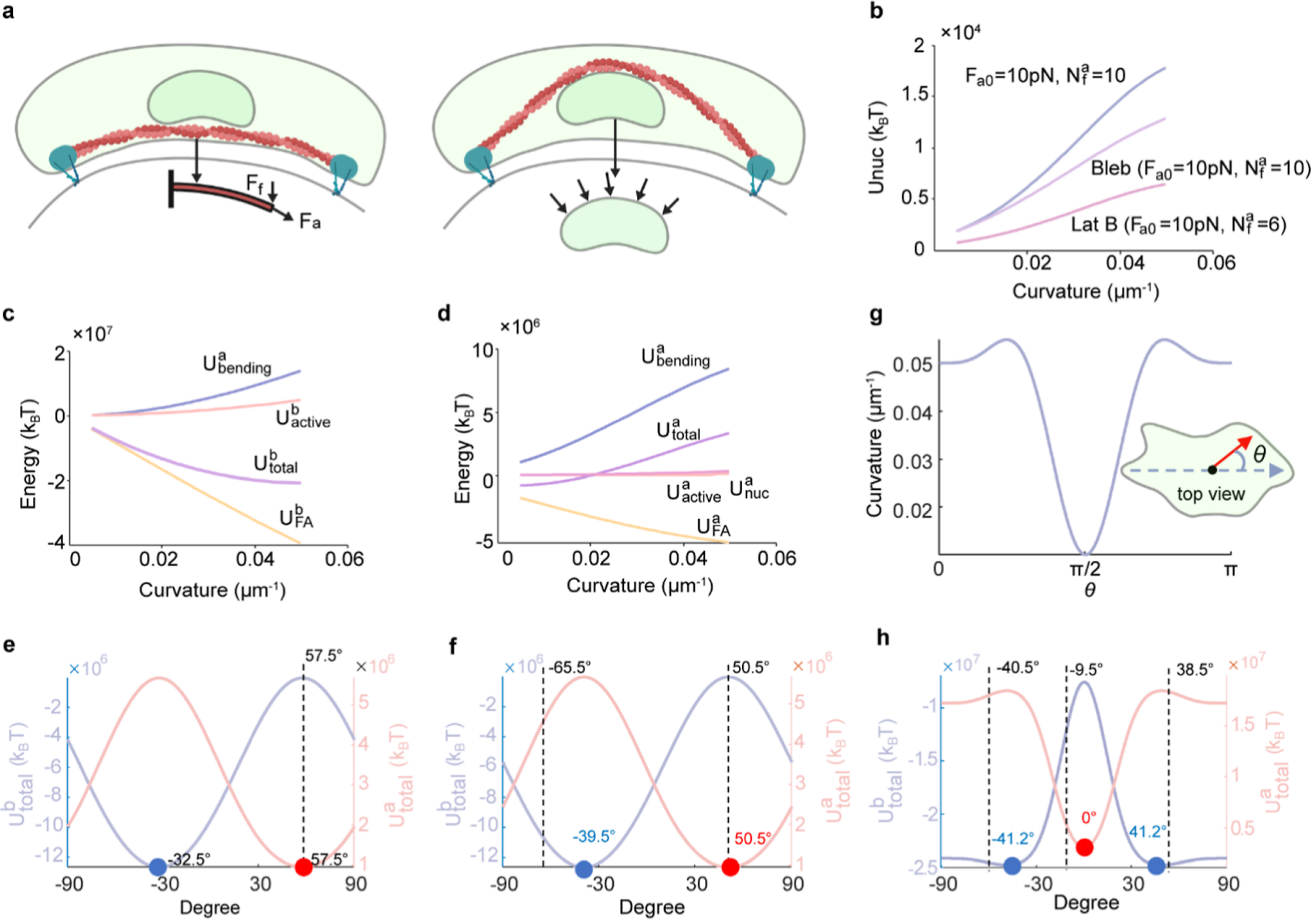
Mechanical model of cell mechanosensing on curved substrates. (a) Schematic illustration of the two-layer mechanical model. The basal layer comprises SFs aligned along the substrate curvature, while apical SFs spanning over the nucleus generate compressive forces that regulate nuclear deformation. (b) Nuclear strain energy grows with curvature (*F*_*a*0_ = 10 pN, *N*_*f*_^*a*^ = 10). With the treatment of Latrunculin B or Blebbistatin, the apical SFs’ quantity (*N*_*f*_^*a*^) and contractile force (*F*_*a*0_) correspondingly decrease, leading to a reduced nuclear strain energy. (c) Basal-layer energy components versus curvature, showing passive bending, active contraction, and FA clustering determine that high curvature is energetically favored by basal SFs. (d) Energy components of the apical layer as a function of curvature, including SFs bending and active contraction, nuclear compression, and FA binding energies. (e,f) Spatial distributions of basal- and apical-layer stress-fiber (SF) energies on uniaxial-low (e) and uniaxial-high (f) surfaces. Solid dots indicate curvature regions that preferentially favor SF alignment in the basal (blue) or apical (orange) layer. The black dashed lines denote the experimentally measured peak SF orientation. (g) A constructed surface with multiaxial curvatures, where θ denotes the azimuthal angle of a vertical cutting plane rotating around the surface normal at a given point. (h) The spatial distribution of basal- and apical-layer stress-fiber (SF) energies on the surface shown in (g). Solid dots indicate curvature regions that preferentially favor SF alignment in the basal (blue) or apical (orange) layer. The black dashed lines denote the experimentally measured peak SF orientations on surfaces with multiaxial curvatures.

In contrast, apical SFs form an actin cap that spans over and compresses the nucleus (Figure 5a), the corresponding total energy is expressed as

2 $$U_{total}^{a}=U_{bending}^{a}+U_{active}^{a}+U_{FA}^{a}+U_{nuc}$$

In eqs 1 and ([Disp-formula eq2]), the subscripts denote contributions from SFs bending, active contraction, and FA, respectively, while the superscripts *a* and *b* represent apical and basal layers, and *U*_nuc_ stands for the strain energy of the nucleus (refer to the Supporting Information for modeling details, parameter values in Table S1, and validation in Figure S18).

We first demonstrate that the nuclear strain energy increases monotonically with substrate curvature (Figure 5b), indicating that the nucleus experiences greater compression on substrates with higher curvature, as observed in Figure 3c. When either the active contractility or the number of apical SFs is reduced, the nuclear strain energy decreases accordingly. This suggests a weakened mechanosensing response, consistent with the observations in cells treated with blebbistatin or Latrunculin B, where contractility and actin polymerization are disrupted (Figure S12). In Figure 5c, we plot the individual energy components of basal SFs as functions of substrate curvature. As curvature increases, both the bending and active contractile energies rise but at a slower rate than the decrease in FAs binding energy. As a result, *U*_total_^*b*^ decreases with increasing curvature, suggesting that basal SFs tend to align along directions with higher curvature to minimize energy. For apical SFs, the FA-associated energy dominates at low curvature. However, as the curvature increases, the bending energy becomes increasingly significant and eventually outweighs the influence of FAs (Figure 5d). This is due to the increased deflection caused by the nucleus height, which markedly elevates the bending energy of apical SFs. Consequently, apical SFs prefer to align along directions of the minimal curvature. We further validated the model-predicted SFs orientation preferences by directly comparing them with experimental measurements on both uniaxial and multiaxially curved substrates (Figure 5e–h). For the uniaxial-low and uniaxial-high surfaces (Figure 5e,f), the experimentally observed dominant SF orientations (Figure 4d, black dashed lines) show an overall trend consistent with the model-predicted low-energy directions, supporting the idea that SFs preferentially align along energetically favorable orientations. To assess the role of the multiaxial curvature, we constructed a surface whose curvature varies with the azimuthal angle, θ (Figure 5g). Mapping the basal and apical energy landscapes over θ reveals multiple preferred SF orientations, and the angles of energy minima differ between the basal and apical layers (Figure 5h), indicating that multiaxial curvature can promote SF reorientation along multiple, not necessarily orthogonal, directions. Together, our mechanical model reveals that substrate curvature significantly influences apical SF bending, which compresses the nucleus and may elevate the level of Lamin A/C expression. The interplay between curvature-induced SF bending and contractility-regulated FA energy determines the orientation of SFs, promoting alignment along the energy-minimizing directions. This mechanism could enhance cell spreading on substrates with a multiaxial curvature.

### Multiaxial Curvature Engineering on 3D Ultrasoft Microgels and Their Applications in Tissue Engineering

Building on the success of curvature engineering in 2D ultrasoft hydrogels and the pivotal role of topological cues in cellular mechanosensing, we extended our SICE approach to 3D microgels. Injectable microgels offer distinct advantages over bulk gels in tissue engineering, such as bone regeneration, cartilage repair, and wound healing,^18,71^ due to their high surface area-to-volume ratio, tunable porosity, and ability to conform to irregular defects, facilitating cell infiltration, nutrient diffusion, and host tissue integration.^72^ This transition to 3D systems serves two purposes: first, to test the applicability of curvature-mediated effects in a three-dimensional context and, second, to amplify the biomedical relevance of our approach by leveraging injectable microgels for tissue repair.

We fabricated size-controlled microgels using a high-throughput water-in-oil (W/O) microfluidic emulsion technique, producing monodisperse microgels (Figures 6a and S14, Table S2).^72^ Following UV cross-linking and purification, the microgels underwent SICE processing, as validated in 2D bulk gels, involving ethanol dehydration to induce surface buckling and water rehydration to stabilize multiaxial curvatures (Figure 6b,c). Three microgel sizes were fabricated (97.2 μm, 203.8 μm, and 532.4 μm, Figure 6d). Microscopic analysis confirmed that SICE treatment preserved the overall microgel dimensions, with no significant size changes postprocessing (Figures 6d and S15a). Atomic force microscopy (AFM) revealed a slight stiffness increase post-SICE (11% for small microgels and 8% for large microgels), yet the stiffness remained within the ultrasoft range 500–600 Pa (Figures 6d and S15b). Curvature quantification showed an inverse relationship between microgel size and curvature: small, moderate, and large SICE-treated microgels exhibited absolute mean curvatures of 0.040 ± 0.025 μm^–1^, 0.031 ± 0.024 μm^–1^, and 0.017 ± 0.013 μm^–1^, respectively (Figures 6c,e and S15c). This size-dependent curvature gradient—combined with tunable stiffness in a low stiffness range—collectively validates SICE as a robust platform for creating injectable microgels with programmable topological and mechanical cues for soft tissue engineering applications.

**Figure 6:**
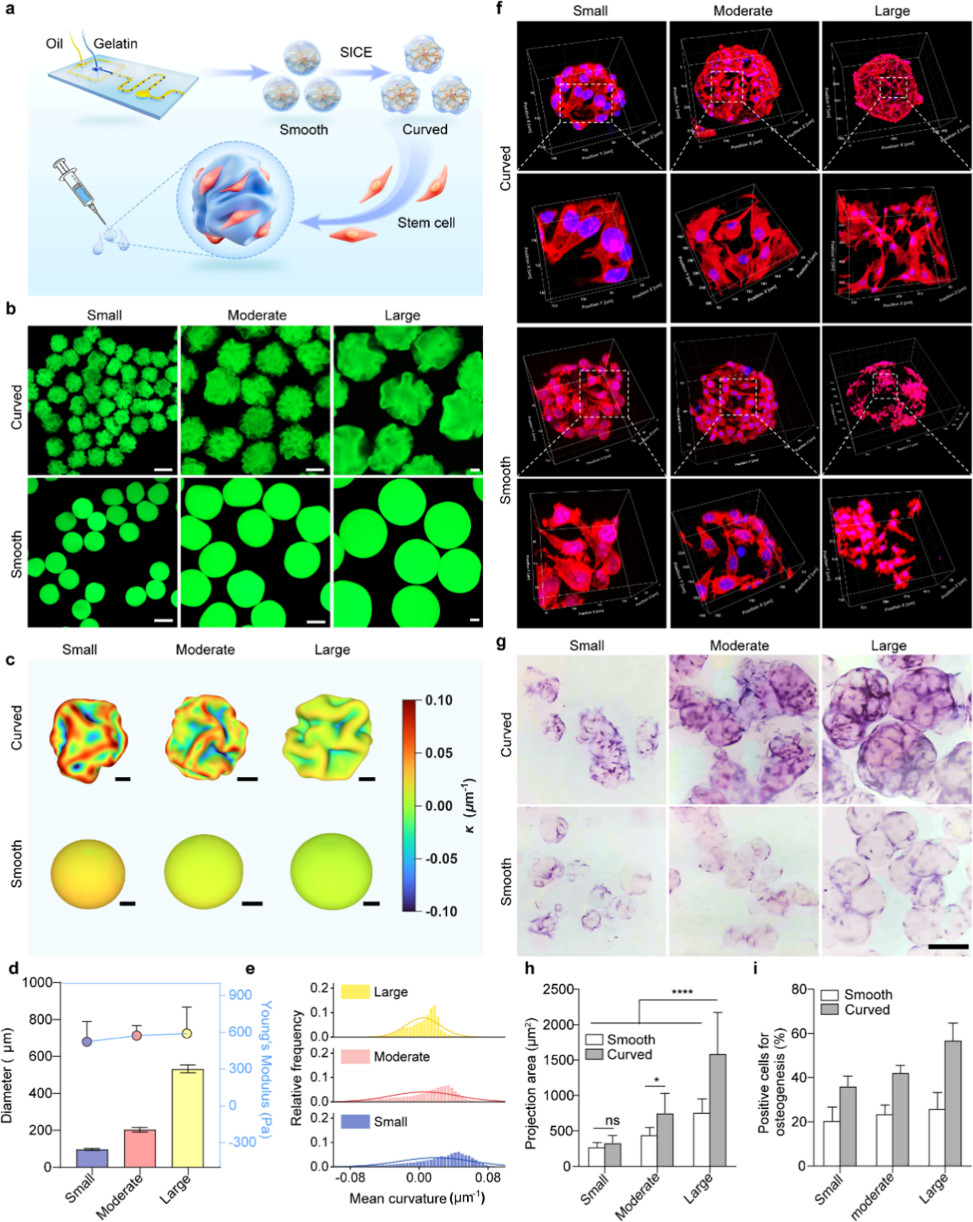
Multiaxial curvature engineering on 3d ultrasoft microgels for stem cell mechanosensing and tissue engineering applications. (a) Schematic illustrating the microfluidic technique for generating ultrasoft microgels with a multiaxial curvature via the SICE process. (b) Fluorescence microscopy images comparing the surface morphology of smooth and SICE-treated multiaxial curvature microgels across different sizes. Scale bar: 100 μm. (c) Color map showcasing the curvature features of 3D microgels with varied curvature conditions. The scale bars indicate 20 μm, 50 μm, and 100 μm from left to right. (d,e) Quantitative analysis of the size (*N* = 30–50), stiffness (*N* = 10), and curvature of 3D microgels (*N* = 3). (f) Immunofluorescence staining of F-actin (red) and nuclei (blue) in hMSCs cultured on 3D microgels with different curvature conditions. (g) Osteogenic differentiation of hMSCs on 3D microgels with varying curvature conditions. Cells were cultured in osteogenic medium for 7 days and stained with alkaline phosphatase (ALP) dye to indicate early osteogenic differentiation. Scale bar: 500 μm. (h–i) Quantification of hMSCs’ spreading area (*N* = 15–20, three technical replicates) and osteogenic differentiation (based on ALP activity) on 3D microgels (*N* = 10–15, three technical replicates).

To evaluate the behavior of hMSCs on these 3D curved microgels, we seeded cells onto both SICE-treated (curved) and untreated (smooth) microgels of varying sizes. Due to the spherical morphology and free motion of the microgels in suspension, cell seeding efficiency was initially low, as cells predominantly sedimented onto the culture dish substrate. To address this, we optimized the seeding protocol: microgels were coincubated with high-density hMSCs in antiadhesive well plates for 2 h to facilitate initial attachment, followed by a 3 min gentle centrifugation to remove unattached cells, and then transferred to low-adhesion plates for 24 h culture. Cells on curved microgels exhibited significantly larger adhesion areas and higher polarization compared to those on smooth microgels, where cells remained predominantly spherical or minimally polarized (Figure 6f,h). This enhanced spreading and alignment mirrors our observations on 2D bulk gels (Section 2.2), confirming that curvature effects persist in 3D microgel systems.

Notably, hMSCs on large microgels with multiaxial curvatures displayed greater spreading and polarization than those on small microgels (Figure 6f,h). This size-dependent behavior arises from both spatial and physical effects of the curvature. On small microgels, smaller groove-like structures (12.9 μm depth, 12.8 μm width; Figure S16) and high circumferential curvatures (>0.07 μm^–1^) restrict physical space and increase cytoskeletal bending energy, inhibiting actomyosin contractility and limiting cell spreading. In contrast, large microgels, with larger grooves (94 μm depth and 73.9 μm width) and lower curvature (0.017 μm^–1^), provide ample space and a mechanically favorable environment for hMSC spreading (Figures 6e and S16). hMSCs exhibit similar adhesion behavior to that on 2D C_100_ hydrogels, with cells adhering and bending along one sidewall of the wrinkle (Figure S17). These results underscore the importance of optimizing curvature scales to match cellular dimensions for effective responses.

To assess the potential of curved microgels in bone tissue engineering, we conducted osteogenic induction experiments, culturing hMSCs on SICE-treated and smooth microgels in osteogenic medium for 7 days. SICE-treated microgels significantly enhanced ALP expression compared to smooth microgels (Figure 6g,i), indicating superior osteogenic differentiation. This aligns with our 2D findings (Section 2.2) and underscores the efficacy of curvature engineering in promoting stem-cell-based bone repair within an injectable, ultrasoft format.

In summary, we successfully extended multiaxial curvature engineering from 2D bulk gels to 3D microgels, enhancing hMSCs adhesion, polarization, and osteogenic differentiation. By decoupling topological cues from substrate stiffness, our approach provides a robust platform for designing injectable biomaterials that recapitulate the biomechanical complexity of native tissues. The ability to tailor microgel size, curvature, and stiffness positions this strategy as a promising tool for regenerative therapies, particularly in soft tissue repair, where stiff scaffolds are impractical.

## Conclusion

Inspired by buckling in biological soft tissues, we developed a SICE strategy to fabricate complex multiaxial curvatures on ultrasoft hydrogels (0.3–4.3 kPa). This approach produced stable surface curvatures spanning 0.007–0.035 μm^–1^—covering most topological scales found in natural soft tissue. This approach revealed that, in ultrasoft microenvironments (<1 kPa), topology—rather than substrate stiffness—serves as the dominant regulator of cellular mechanosensing. We found that cells respond to multiaxial curvatures by reorganizing the spatial distribution of SFs and focal adhesions: basal fibers align along high-curvature directions to activate Rho-mediated contractility and generate intrinsic tension; apical fibers align along low-curvature directions to minimize bending energy. This strategy enables cells on ultrasoft interfaces to actively maintain mechanical tension, thereby enhancing cell nuclear stress level, spreading, and differentiation.

These findings complement the traditional stiffness-centric paradigm of mechanobiology, indicating that in compliant environments such as soft tissues, cells can prioritize decoding geometric topological cues to sustain tension and guide fate decisions. Furthermore, the successful translation of this approach to 3D injectable microgels underscores its potential for regenerative applications. This study not only provides an innovative fabrication tool but also proposes a distinct biomaterial design principle: engineering multiaxial curvatures into ultrasoft scaffolds can create microenvironments capable of actively directing stem cell functions.

There are several limitations in the current study. These include insufficient validation of curvature stability in vivo and the need for further optimization of the mechanical model. Additionally, although our study focuses on the cytoskeletal and nuclear execution of curvature sensing, the initial molecular sensors that translate multiaxial geometric cues into biochemical signals remain to be identified. Future investigations should explore the potential role of canonical membrane curvature sensors, such as BAR-domain proteins,^31,73^ in this process. Determining whether and how these proteins engage with our engineered multiaxial curvatures will connect our biophysical observations to a more comprehensive molecular pathway of topographic mechanotransduction. Future research will also focus on verifying in vivo therapeutic efficacy and examining its universality across other cell types. By gaining a deeper understanding of topological curvature as a form of “mechanical language,” we will be able to design biomaterials that engage directly and efficiently with cells, opening new pathways in regenerative medicine and fundamental biological research.

## Experimental Section

### Fabrication of 2D Wrinkled Hydrogels via SICE

2D wrinkled hydrogels were prepared using a SICE strategy. GelMA was first dissolved in phosphate-buffered saline (PBS) to a final concentration of 4.5% (w/v) and then mixed with lithium phenyl-2,4,6-trimethylbenzoylphosphinate (LAP, 20 mg/mL in PBS, Macklin, China) at a volumetric ratio of 100:12 (GelMA: LAP). After thorough mixing, 29 μL of the prepared solution was pipetted onto circular glass coverslips (12 mm diameter, Solarbio, China) and evenly spread, followed by 30 s photo-cross-linking under 365 nm UV light (550 mW/cm^2^, ZIGOO, China) to form flat hydrogels. The resulting hydrogels were then dehydrated by immersion in PBS/ethanol mixtures of varying ethanol volume fractions (100%, 90%, 80%, 70%, 60%, and 0%) for 20 min. After gently blotting excess ethanol solvent from the surface with tissue paper, the hydrogels were allowed to swell in PBS for 10 min to trigger wrinkles’ generation on their surface. To stabilize the formed wrinkle patterns, the hydrogels were post-cross-linked in 1% glutaraldehyde (Macklin, China) PBS solution for 2 h. The cross-linked hydrogels were subsequently washed three times with PBS and incubated in 0.1% gelatin (in PBS) for 1 h to quench residual aldehyde groups. Finally, the hydrogels were soaked in PBS for 24 h to remove unreacted small molecules prior to cell culture experiments.

### Fabrication of Curved 3D Microgels via Microfluidics and SICE

First, a 4.5% (w/v) GelMA solution containing LAP in PBS was prepared, as previously described. The obtained solution was then introduced into a microfluidic chip, where it was sheared into droplets by a continuous phase of paraffin oil (MedChemExpress, USA) containing 1% ABIL EM 90 (v/v, Degussa, Germany) and 1% polyglycerol-polyricinoleate (PGPR, v/v, MedChemExpress, USA). The resulting gelatin droplets were cross-linked by exposure to 365 nm UV light (1 min), forming stable microgels. The designed microfluidic chip is illustrated in Figure S14 and is fabricated using a 3D printing process (Form 3B+, Formlabs). Through flexibly adjusting the flow rates of the polymer and oil phases, the microgels with different diameters were fabricated (Table S2). The cross-linked microgels were collected and repeatedly washed with *n*-hexane (Sigma, USA) to remove residual paraffin oil and surfactants, followed by vacuum drying to eliminate the residual solvent. To induce curvature, the microgels were subjected to the SICE process following a protocol similar to that described above. Briefly, microgels were dehydrated by immersion in ethanol for 14 days and then transferred to PBS to induce swelling and the formation of curved morphology. The curved microgels were subsequently incubated in 1% glutaraldehyde and 0.1% gelatin solutions and PBS at 4 °C for 24 h to remove residual small molecules before further use.

### Cell Culture

The hMSCs used in this study are a commercially available cell line (ATCC). No human participants or animal subjects were directly involved in the research, and therefore specific ethics approval was not required. The stem cells were cultured in MesenPRO RS Medium (Gibco, 12746012) with supplementation of 1% penicillin/streptomycin (Gibco, 15140122) and 2 mmol l-glutamine (Gibco, 25030081), under standard conditions of 37 °C and 5% CO_2_. The osteogenic differentiation medium (Gibco, A1007201) was obtained from Thermofisher Scientific, USA. According to the standard protocol, hMSCs were passaged at least once a week, and passages 5 to 9 were used for all experiments. For each experiment, cells from the same passage were randomly allocated to different experimental groups (C_100_, C_70_, FLAT, etc.). All groups were processed in parallel to minimize the batch effects. For seeding cells on 2D wrinkled hydrogels, hydrogel samples were first sterilized under UV light for 30 min and transferred into 24-well plates. hMSCs were then detached and seeded onto the hydrogels at a density of 6,000 cells/well, followed by overnight incubation to ensure cell attachment before subsequent experiments. For seeding cells on 3D microgels, a similar procedure was followed, with a few modifications to improve cell attachment efficiency on the curved microgel surfaces. Cells were suspended at a higher concentration (500,000 cells/mL), and Polydimethylsiloxane (PDMS, Dow Corning, SYLGARD 184 Silicone Elastomer Kit) coating was applied to the bottom of the well to prevent nonspecific adhesion. Meanwhile, to reduce sedimentation-induced uneven attachment, the culture medium was gently agitated every 30 min for the initial 2 h (three times in total) after seeding.

### Immunostaining Staining

Cells cultured on the hydrogels were rinsed with PBS (Thermofisher Scientific, 10010023) and fixed in 4% paraformaldehyde (Sigma, 1209228) for 15 min at room temperature. Following fixation, cells were permeabilized with 0.25% PBST solution (Triton X-100 (Sigma, 2315025) PBS solution) for 5 min and then blocked with 1% BSA-0.1% PBST solution (Bovine serum albumin, Sigma, 2329362) for 45 min at room temperature. For immunostaining, the following primary antibodies were applied overnight at 4 °C in blocking buffer to stain corresponding proteins: mouse monoclonal Alexa Fluor 488-conjugated vinculin antibody (Invitrogen, 14977782, 1:50), mouse monoclonal Lamin A/C antibody (Invitrogen, MA535284, 1:50), rabbit polyclonal YAP antibody (Cell Signaling, 4912S, 1:50), goat polyclonal MYPT1Antibody (Invitrogen, PA5-142374, 1:50), and rabbit monoclonal Anti-Sp7/Osterix antibody (Abcam, ab209484, 1:50). After washing twice with PBS, cells were incubated with secondary antibodies (Invitrogen, Goat anti-Mouse IgG Alexa Fluor 488 A-11029 or Goat anti-Rabbit Alexa Fluor 568 A-11011 or Donkey anti-Goat IgG Alexa Fluor 594 A-11058, 1:500 dilution) for 1 h at room temperature in the dark. Lastly, cells were incubated with Hoechst 33342 (MedChemExpress, HY15559, 1:1000) and Phalloidin-iFluor 488/647 (Abcam, ab176753/ab176759, 1:1000) for nuclear and F-actin staining and imaged with a laser confocal microscope (LSM 980, Zeiss, Germany). For image analysis, cells that were out of focus, undergoing division, or at the edge of the field of view were excluded from quantification.

### Inhibition and Activation Experiment of Cells

Cells were treated with inhibitors or activators during seeding and cocultured for 24 h. Rho activation was induced using CN03 (Cytoskeleton, #CN03-B) at a concentration of 10 μg/mL. ROCK inhibition was achieved with Y27632 (MedChemExpress, HY-10071) at 10 μM. Actin polymerization was blocked by Latrunculin B (MedChemExpress, HY-101848) at 0.2 μM, while myosin activity was inhibited with blebbistatin (MedChemExpress, HY-13813) at 20 μM.

### Statistical Analysis

All data are presented as mean ± standard deviation (sd) except where specifically indicated. Statistical comparisons between groups were assessed using one-way ANOVA followed by Tukey’s multiple comparison test. A *P*-value less than 0.05 was considered statistically significant (*P < 0.05; **P < 0.01; ***P < 0.001; ****P < 0.0001). Analyses were conducted using GraphPad Prism version 8.

## Supplementary Material



## Notes

Hydrogel preparation; morphology characterization; mechanical characterization; TFM measurement; generation and processing of bulk RNA-seq data; cell differentiation; and mechanical modeling (PDF)
