# Supplementary material for: Topology Outweighs Stiffness: Self-Reinforced Cell Mechanotransduction via Multiaxial Curvature Engineering of Ultrasoft Hydrogels
Source: ACS Nano. 2026 Feb 24;20(9):7679–92. doi: 10.1021/acsnano.5c19367 (PMC12981023; doi:10.1021/acsnano.5c19367)
Supplement: Supplementary file 1 [file nn5c19367_si_001.pdf]

Supporting information

Topology Outweighs Stiffness: Self-Reinforced Cell Mechanotransduction via Multiaxial Curvature Engineering of Ultrasoft Hydrogels

Yong Hou[1#], Xinhao Hu[1#], Cheng Qian[2], Wenyan Xie[3], Linjie Ma[1], Luyao Zhang[1], Xiaomei Han[3], Youhua Tan[3], Yuan Lin[4], Chao Fang[2*], Zhiqin Chu[1,5,6*]*

1. Department of Electrical and Electronic Engineering, The University of Hong Kong, Pok Fu Lam, Hong Kong, China
2. School of Science, Harbin Institute of Technology, Shenzhen, Guangdong, China.
3. Department of Biomedical Engineering, Hong Kong Polytechnic University, Kowloon, Hong Kong, China
4. Department of Mechanical Engineering, The University of Hong Kong, Pok Fu Lam, Hong Kong, China
5. School of Biomedical Sciences, The University of Hong Kong, Pok Fu Lam, Hong Kong, China
6. School of Biomedical Engineering, The University of Hong Kong, Pok Fu Lam, Hong Kong, China

* Corresponding authors:

Prof. Dr. Yuan Lin, Email: ylin@hku.hk

Prof. Dr. Chao Fang, Email: fangchao@hit.edu.cn

Prof. Dr. Zhiqin Chu, Email: zqchu@eee.hku.hk

\# The authors contribute equally to this work.

Methods

Synthesis of methacrylated gelatin (GelMA)

Methacrylated gelatin was synthesized using a modified protocol. Briefly, 5 g of type B, 225g bloom bovine skin gelatin (Sigma, USA) was dissolved in 50 mL of phosphate-buffered saline (PBS) at 60 °C under constant stirring. 1 mL methacrylic anhydride (Sigma, USA) was then added dropwise to the gelatin solution and allowed to react for 2 hours. The reaction was quenched by adding 100 mL of pre-warmed PBS (40 °C). The resulting mixture was transferred to a dialysis membrane (14 kDa, BKMAM, China) and dialyzed against distilled water at 37 °C for 4 days. The final product was lyophilized to obtain a white porous foam and stored under argon at -20 °C until further use.

Preparation of alginate hydrogel, matrigel, and polyacrylamide gel.

Alginate hydrogels were prepared via ionic crosslinking. Briefly, 80 μL of sodium alginate solution (1% w/v in deionized water; Sigma) was pipetted onto a glass-bottom dish. Subsequently, 80 μL of calcium chloride solution (1% w/v; Sigma) was gently added dropwise to the alginate droplet to initiate crosslinking. The gel was allowed to polymerize at room temperature for 5–10 min. Matrigel hydrogels were formed by temperature-induced gelation. A volume of 160 μL of Matrigel (NMO-G, Novoprotein) was carefully dispensed into a glass-bottom dish and incubated at 37 °C for 30 min to allow complete polymerization. Polyacrylamide hydrogels were synthesized via free-radical polymerization. A pre-gel solution was prepared by mixing 75 μL of acrylamide solution (40% w/v; Bio-Rad), 30 μL of bis-acrylamide solution (2% w/v; Bio-Rad), and 895 μL of deionized water in a 1 mL vial, followed by gentle vortex mixing. Immediately before use, 10 μL of ammonium persulfate (APS, 10% w/v; Sigma) and 1 μL of N,N,N′,N′-tetramethylethylenediamine (TEMED; Sigma) were added to the pre-gel mixture and mixed thoroughly. Then, 160 μL of the final polymer solution was transferred to a glass-bottom dish and allowed to polymerize for 1 h at room temperature.

Hydrogel morphology characterization

The morphology of the fabricated 2D wrinkled hydrogels and 3D curved microgels was characterized via 3D reconstruction using laser confocal microscopy (LSM 980, Zeiss, Germany). Briefly, the hydrogels were first stained with 0.2 mg/mL Fluorescein isothiocyanate PBS solution for 1 hour. Then, the samples were transferred to confocal dishes and 3D reconstructed using a laser scanning confocal microscope with a Z-stack interval of 2 μm. The wrinkle depth and width were quantified using the orthogonal view function in ZEN 3.9 software (Zeiss, Germany).

Mean curvature were calculated to perform quantitative analysis how curvature of hydrogel surface features adjusts hMSCs mechanics. For 2D hydrogels, the two principal curvatures (K_1 and K_2) were approximated via the "Kappa-Curvature Analysis" plugin in ImageJ along two orthogonal cross-sections which were extracted using the Orthogonal Image function in ZEN 3.9 software. For 3D microgels, mean curvature mapping was performed using Imaris (version 10.1) and MeshLab (version 2023.12). Specifically, the 3D reconstructed microgel was first imported into Imaris to generate a surface rendering. The resulting surface mesh was then exported to MeshLab, where the "Compute Curvature Principal Directions" function was used to calculate the mean curvature distribution.

Hydrogel mechanical characterization

Mechanical characterization of the hydrogels was carried out using atomic force microscopy (AFM, Bruker, Germany). A tipless cantilever functionalized with a 10 μm polystyrene bead was used to measure force–displacement curves on the hydrogel surface. The Young's modulus was extracted by fitting the curves with the Hertz/Sneddon model. For each sample, three distinct regions on the wrinkled surface were randomly selected, and each region was probed using a 3 × 3 array of indentation points.

TFM measurement

Wrinkled GelMA hydrogels embedded with 0.5 μm fluorescent carboxylated polystyrene beads (Red fluorescence, Sigma, L3280) were prepared using a modified protocol based on the method described above. Briefly, 5 μL of fluorescent beads were mixed with 1 mL of GelMA/LAP prepolymer solution and homogenized thoroughly. The mixture was then pipetted onto glass coverslips and evenly spread. Photo-crosslinking was performed by exposure to 365 nm UV light (550 mW/cm², ZIGOO, China) for 30 s.

After crosslinking, hydrogels were sequentially treated with ethanol, PBS, 1% glutaraldehyde and 0.1% gelatin PBS solutions to generate wrinkled topology on the surface, then immersed in PBS at 4 °C for 24 h before use.

Cells were seeded on the hydrogels and incubated overnight. Before imaging, cells were stained with Calcein-AM (Sigma, C1359), and fluorescence images of both cells and beads were captured to record the initial positions of the beads beneath spread cells. Cells were then lysed using 0.5% Sodium dodecyl sulfate (SDS, Invitrogen, 1742382) for 10 minutes, followed by acquisition of post-lysis bead images. Bead displacement analysis was performed using Image J. First, image drift was corrected using the “Align slices in stack” plugin. Then, the “Particle Image Velocimetry” plugin was used to compute bead displacement fields across the cell area. The displacement vectors were reconstructed and visualized using the “Plot Particle Image Velocimetry” plugin to generate traction maps.

hMSC differentiation

hMSCs were seeded on hydrogel substrates in growth medium as described above. After 24 hours of culture, the medium was replaced with standard osteogenic induction medium and maintained for 4-7 days, with medium changes every 3 days.

On 2D hydrogels, osteogenic differentiation was evaluated on day 4 by immunostaining for Osterix（see *Immunofluorescence* section for details）, which is a transcription factor

critical for osteoblast differentiation and mineralization. The degree of differentiation was determined by analyzing its subcellular localization (nuclear vs. cytoplasmic).

For 3D microgels, osteogenic activity was assessed on day 7 by alkaline phosphatase (ALP) staining. Cells were fixed in 4% paraformaldehyde for 5 minutes and stained with Pluripotent Stem Cell Alkaline Phosphatase Color Development Kit (Beyotime, C3250S) following the manufacturer's protocol, followed by Hoechst 33342 counterstaining. Differentiation efficiency was quantified by calculating the ratio of ALP-positive to ALP-negative cells in at least five randomly selected fields (20× objective) across microgels of varying curvature.

Generation and processing of bulk RNA-seq data

Total RNA was isolated from hMSC cells using TRIzol Reagent (Invitrogen, USA) according to the manufacturer's instructions. The RNA quality was verified using Agilent 2100 Bioanalyzer (Agilent Technologies, USA). The cDNA libraries were prepared and sequenced on a NovaSeq X Plus platform (Illumina, Inc., USA).

Raw reads were quality-trimmed and adapter-filtered using fastp (v0.24.0) (*1*). Read quality was assessed using FastQC (v0.12.1) (https://github.com/s-andrews/FastQC), followed by alignment to human reference genome (GRCh38/hg38) with HISTA2 (v2.2.1) (*2*). Quantification of the clean reads was performed using featureCounts (*3*). Differential gene expression analysis was conducted using DESeq2 (*4*), with log2 fold change > 1 and adjusted P value < 0.05. The differentially expressed genes were subsequently subjected to Gene Ontology (GO) enrichment analysis (PMID: 10802651).

Mechanical model

Based on experimental observations, we model the apical and basal stress fibers (SFs) independently with the nucleus positioned in between. For the basal layer, individual SF is regarded as an elastic beam that symmetrically conforms to curved substrate with curvature K (Fig. 5A). To simplify the analysis, we approximate each SF as a clamped

beam with half the actual fiber length. Under this assumption, the true profile of arc-shaped basal SFs is given by

$$w_{arc}^{b}(s) = \frac{1-\cos(Ks)}{K} \tag{S1}$$

where s denotes the arc-length coordinate along the fiber. Note that the SFs are anchored via focal adhesions (FAs), which transmit forces akin to concentrated loads rather than bending moments. Accordingly, in our model, the FA-generated force is treated as a concentrated load F_f^b applied at the free end of a clamped beam. The resulting beam deflection is then expressed as

$$w^{b}(s) = F_f^b \frac{s^2(3L_0^b - s)}{6EI} \tag{S2}$$

where L_0^b is the half-length of a basal SF and EI represents its bending stiffness. To determine the amplitude of F_f^b, we minimize the least-squares deviation between the beam deflection $w^b(s)$ and the true profile $w_{arc}^b(s)$

$$\min_{F_f^b} \int_0^{L_0^b} \left(w_{arc}^b(s) - w^b(s)\right)^2 ds. \tag{S3}$$

Taking the derivative of the objective function with respect to F_f^b and setting it to zero yields the optimal force amplitude

$$F_f^b = \frac{\int_0^{L_0^b} \frac{s^2(3L_0^b - s)}{6EI} \cdot \frac{1-\cos(Ks)}{K}\, ds}{\int_0^{L_0^b} \left(\frac{s^2(3L_0^b - s)}{6EI}\right)^2 ds} \tag{S4}$$

The error between the prescribed arc profile $w_{arc}^b(s;K)$ and the Euler–Bernoulli beam shape $w^b(s;K)$ can be determined with the L2-based RMS fitting method

$$\varepsilon_{\text{abs}}(K) = \sqrt{\frac{1}{L_0^b} \int_0^{L_0^b} \left[w_{arc}^b(s;K) - w^b(s;K)\right]^2 ds} \tag{S5}$$

$$\varepsilon_{\text{rel}}(K) = \frac{\varepsilon_{\text{abs}}(K)}{\sqrt{\frac{1}{L_0^b} \int_0^{L_0^b} w_{arc}^{b\,2}(s;K)\, ds}} \tag{S6}$$

The errors remain modest across the entire curvature range considered (Fig. S18),

supporting that the beam approximation is quantitatively accurate and does not affect the qualitative trends or main conclusions.

The total bending energy stored in the basal SFs is then calculated from the Euler-Bernoulli beam energy functional

$$U_{bending}^{b} = 2N_f^b \times \frac{1}{2}\int_0^{L_0^b} EI\left(\frac{d^2w^b}{ds^2}\right)^2 ds \tag{S7}$$

where N_f^b is the number of basal SFs, and the coefficient 2 accounts for the symmetry of the beam configuration. Note that Eq. (S2) gives

$$\frac{d^2w^b}{ds^2} = \frac{F_f^b}{EI}\left(L_0^b - s\right). \tag{S8}$$

Therefore, we have

$$U_{bending}^{b} = N_f^b \frac{F_f^{b^2} L_0^{b3}}{3EI}. \tag{S9}$$

As reported in (*5*), increased substrate curvature enhances myosin activation within SFs, indicating a positive correlation between curvature and the active contractile force generated by SFs. Based on this observation, we assume the active force to scale linearly with curvature K as

$$F_a = F_{a0}\frac{K}{K_0} \tag{S10}$$

where F_{a0} is the reference contractile force corresponding to a baseline curvature K_0. The corresponding axial strain energy of basal SFs induced by the active contractility is evaluated as

$$U_{active}^{b} = 2N_f^b \int_0^{L_0^b} \frac{F_a^2}{2EA_f} ds = N_f^b \frac{F_a^2 L_0^b}{EA_f}. \tag{S11}$$

Here, individual SF is assumed to be a curved rod subjected to an approximately uniform axial force F_a, and EA_f is the axial stiffness of a stress fiber.

According to previous studies (*6*), mechanical loading promotes the growth and maturation of FAs. Inspired by this, we model the energy associated with clustered FA formation under a total force $F_{tot}^{b}=\sqrt{{F_f^b}^2+F_a^2}$ as

$$U_{FA}^{b}=-\ 2N_f^b k F_{tot}^{b} N_{mol}\Delta G_{bind} \tag{S12}$$

where k is the clustering coefficient of adhesion molecules, N_{mol} is the number of molecules within per FA cluster and ΔG_{bind} represents the energy reduction per adhesion bond formed. Hence, the total energy of the basal SFs under a given curvature is expressed as the sum of bending energy, active contractile energy, and adhesion energy

$$U_{total}^{b}=U_{bending}^{b}+U_{active}^{b}+U_{FA}^{b} \tag{S13}$$

For the apical layer, note that one end of the SF is clamped at the top of the nucleus, while the other is attached to the curved substrate. To model the beam deflection, we have to modify the arc profile in Eq. (S1) by introducing a linear correction term accounting for the nuclear height h_n. The modified expected arc-shaped profile becomes

$$w_{arc}^{a}(s)=\frac{1-cos(Ks)}{K}+\frac{sh_n}{L_0^a}. \tag{S14}$$

This ensures zero deflection at the clamped end and a total vertical offset of h_n at the free end. As in the basal layer, the deflection of the apical SF under the point load F_f^a is expressed as

$$w^{a}(s)=F_f^a\frac{s^2(3L_0^a-s)}{6EI}. \tag{S15}$$

The optimal force amplitude F_f^a is then obtained by minimizing the least-squares deviation between $w_{arc}^{a}(s)$ and $w^{a}(s)$

$$F_f^a=\frac{\int_0^{L_0^a}\frac{s^2(3L_0^a-s)}{6EI}\cdot\left(\frac{1-\cos(Ks)}{K}+\frac{shn}{L_0^a}\right)ds}{\int_0^{L_0^a}\left(\frac{s^2(3L_0^a-s)}{6EI}\right)^2 ds}. \tag{S16}$$

The relative error is as modest as that of the basal layer (Fig. S18). The total load applied to the free end of the apical SF is then given by $F^a_{tot} = \sqrt{F_f^{a2} + F_a^2}$. The energy contributions from apical SF bending, active contraction, and FA growth/maturation could be obtained identically as Eqs. (S7)(S9)(S10)

$$U^a_{bending} = N^a_f \frac{F_f^{a2} L_0^{a3}}{3EI},\ U^a_{active} = N^a_f \frac{F_a^2 \cdot L_0^a}{EA_f}\ ,\ U^a_{FA} = -\ 2N^a_f k F^a_{tot} N_{mol} \Delta G_{bind}.$$

Here, N^a_f is the number of apical SFs, L^a_0 is the half-length of an apical SF, F^a_f is the concentrated load inducing beam bending.

Since apical SFs span across and compress the nucleus, resulting nuclear strain energy, we treat the nucleus as a neo-Hookean cylinder with cross-section area $A_n = \pi r_{nuc}^2$, where r_{nuc} is the nucleus radius. Nuclear compression results primarily from the bending load F^a_f and active contractile force F_a, projected perpendicularly to the nuclear surface. The angle of SF orientation at the free end is

$$sin\alpha = \left.\frac{dw^a_{arc}}{ds}\right|_{s=L^a_0} = sin\left(KL^a_0\right) + \frac{h_n}{L^a_0}. \tag{S17}$$

Thus, the total compressive force exerted on the nucleus is

$$F_{\text{com}} = 2N^a_f(F^a_f + F_a sin\alpha) \tag{S18}$$

The Helmholtz free-energy density (strain-energy density) of the nucleus is written as

$$W = \frac{\mu_{nuc}}{2}(I_1 - 3) \tag{S19}$$

where μ_{nuc} is the nuclear shear modulus, $I_1 = \text{tr}(\mathbf{C})$ is the first invariant of $\mathbf{C} = \mathbf{F}^T \mathbf{F}$, and the incompressibility constraint imposes $J = \det\mathbf{F} = 1$. $\mathbf{F}$ is the deformation gradient tensor. Under predominantly uniaxial deformation with axial stretch ratio λ, the lateral stretches satisfy $\lambda_\perp = \lambda^{-1/2}$, giving

$$I_1 = \lambda^2 + 2\lambda_\perp^2 = \lambda^2 + \frac{2}{\lambda}. \tag{S20}$$

Substituting into Eq. (17) yields

$$W(\lambda) = \frac{\mu_{nuc}}{2}\left(\lambda^2 + \frac{2}{\lambda} - 3\right). \quad \text{(S21)}$$

The stretch ratio λ could be determined by the equivalence of axial nominal stress (force normalized by the reference cross-sectional area A_n) $\sigma = \frac{F_{\text{com}}}{A_n} = \frac{\partial W}{\partial \lambda} = \mu_{nuc}$ $\left(\lambda - \frac{1}{\lambda^2}\right)$. The corresponding strain energy stored in the nucleus is

$$U_{nuc} = \frac{1}{2} A_n h_n \mu_{\text{nuc}} \left(\lambda^2 + \frac{2}{\lambda} - 3\right) \quad \text{(S22)}$$

In summary, the total energy of the apical SF-nucleus system is given by

$$U_{total}^{a} = U_{bending}^{a} + U_{active}^{a} + U_{FA}^{a} + U_{nuc} \quad \text{(S23)}$$

All parameters used in the model are summarized in Table S1.

Curvature of surfaces

UniAxial-low and UniAxial-high are cylindrical geometries (quasi-uniaxial curvatures) with principal curvatures $K_{\parallel} = 0$ and $K_{\perp} = 1/R$. The orientation-dependent normal curvature used in the model is

$$K(\theta) = K_{\parallel} \cos^2(\theta - \theta_0) + K_{\perp} \sin^2(\theta - \theta_0) = \frac{1}{R} \sin^2(\theta - \theta_0) \quad \text{(S24)}$$

where $\theta \in [-90°, 90°]$ and θ_0 denotes the axial direction ($\kappa = 0$). According to our experimental measurements, we set $\theta_0 = 57.5°$ and $K_{\perp} = 0.011\mu m^{-1} (R = 90.9\mu m)$ for UniAxial-low surface. Similarly, we set $\theta_0 = 50.5°$ and $K_{\perp} = 0.018\mu m^{-1}$ $(R = 55.6\mu m)$, for UniAxial-high surface.

Given the unavailability of the exact curvatures of the multiaxial surface, a user-defined orientation-dependent curvature profile, $K_{\text{multi}}(\theta)$, was introduced to generate more complex energy landscapes and to test the model's ability to predict multi-peak alignment. We first defined a dimensionless shape kernel

$$g(\theta) = \cos^{\alpha}\theta \left(A + B\cos(2\theta)\right) \quad \text{(S25)}$$

and then obtained the curvature function via linear scaling and shifting:

$$K_{\mathrm{multi}}(\theta) = \mathrm{scale} \cdot g(\theta) + \mathrm{offset}. \tag{S26}$$

The parameters used in this study were $\alpha = 4$, $A = 0.5$, $B = -1.6$, scale=0.0365, and offset=0.05. These parameter units are consistent with the model input (K_{multi} in μ m^{-1}), allowing $K_{\mathrm{multi}}(\theta)$ to be directly used to compute $E_{\mathrm{basal}}(\theta)$and $E_{\mathrm{apical}}(\theta)$.

Table S1. Value of parameters adopted in our model.

**Physical meaning**	**Parameter**	**Value**	**Unit**
Number of basal SFs	N_f^b	40	-
Half-length of basal SFs	L_0^b	20	μm
Number of apical SFs	N_f^a	10	-
Half-length of apical SFs	L_0^a	40	μm
Axial stiffness of SFs	EA_f	24.7	pN
Bending stiffness of SFs	EI	5×10^4	pN·μm^2
FA clustering coeffcient	k	2	pN^{-1}
Molecules per FA cluster	N_{mol}	150	-
Binding free energy per molecule	ΔG_{bind}	4.1	pN·nm
Initial nuclear height	h_n	8	μm
Nuclear radius	r_{nuc}	10	μm
Nuclear shear modulus	μ_{nuc}	1000	Pa
Reference active force at reference curvature κ_0	F_{a0}	10	pN
Reference curvature for normalization	K_0	1/50	μm^{-1}

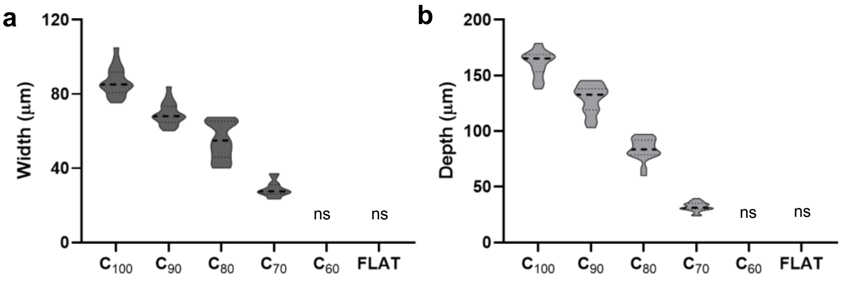

Figure S1. Topology characterization of hydrogels under different ethanol treatment. (a) The width and (b) the depth of the formed wrinkles.

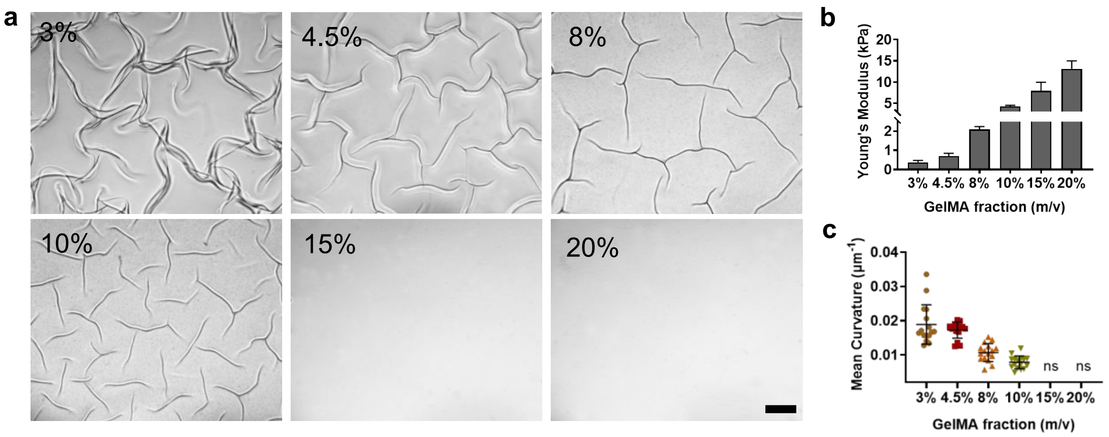

Figure S2. Wrinkled hydrogels fabricated with different gelation fractions. (a) Optical images of fabricated hydrogels with 3%, 4.5%, 8%, 10%, 15%, 20% fraction (w/v); (b) Young's modulus of fabricated hydrogels; (c) The curvedness of the formed wrinkles; the scale bar indicates 200 μm.

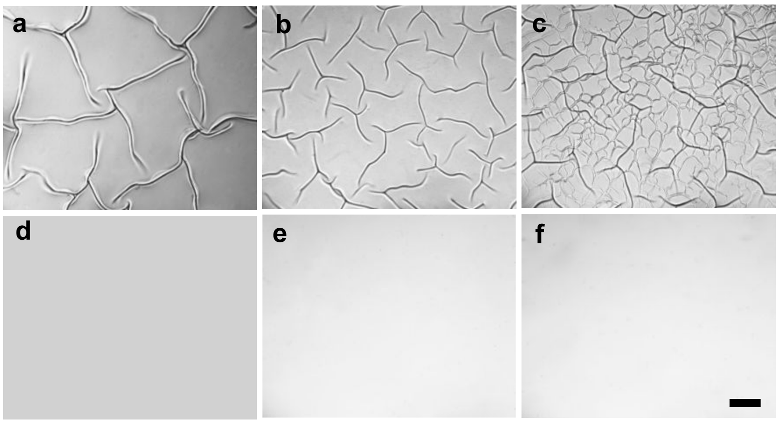

Figure S3. The image of hydrogels treated by different solvents. (a) Methanol; (b) isopropanol;(c) acetone; (d) acetonitrile; (e) DMF; (f) DMSO; the scale bar indicates 200 μm.

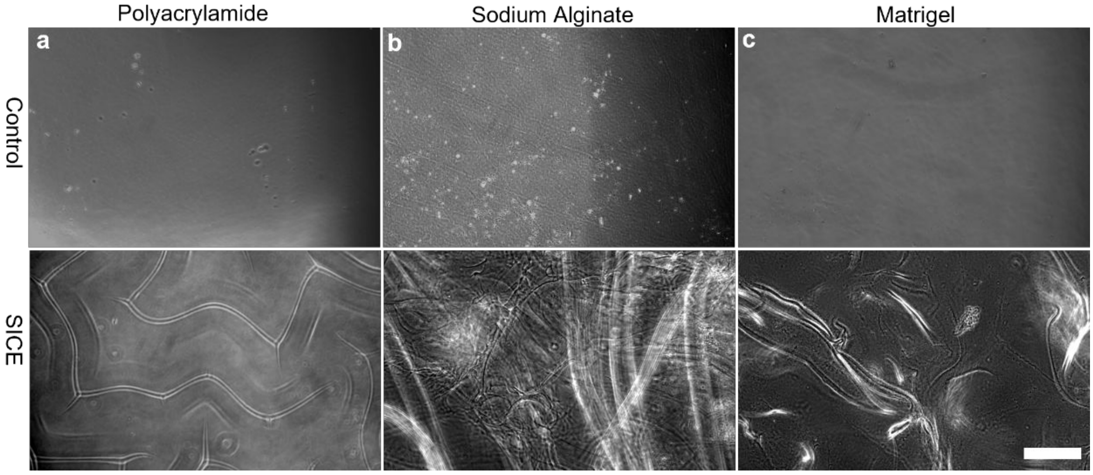

Figure S4. Fabrication of wrinkled hydrogels with different polymers via SICE. a) Polyacrylamide hydrogel (3% w/v); b) Sodium Alginate (0.5% w/v); Matriagel (1.3% w/v). Scale bar: 100 μm.

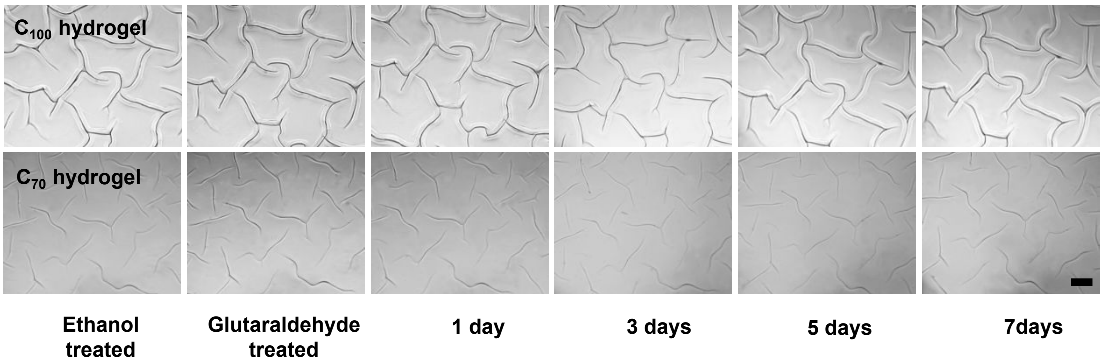

Figure S5. Stability test of the ultrasoft hydrogel with curvatures. Optical images of wrinkled hydrogels incubating at 37℃ for 7 days in cell culture medium; the scale bar indicates 200 μm.

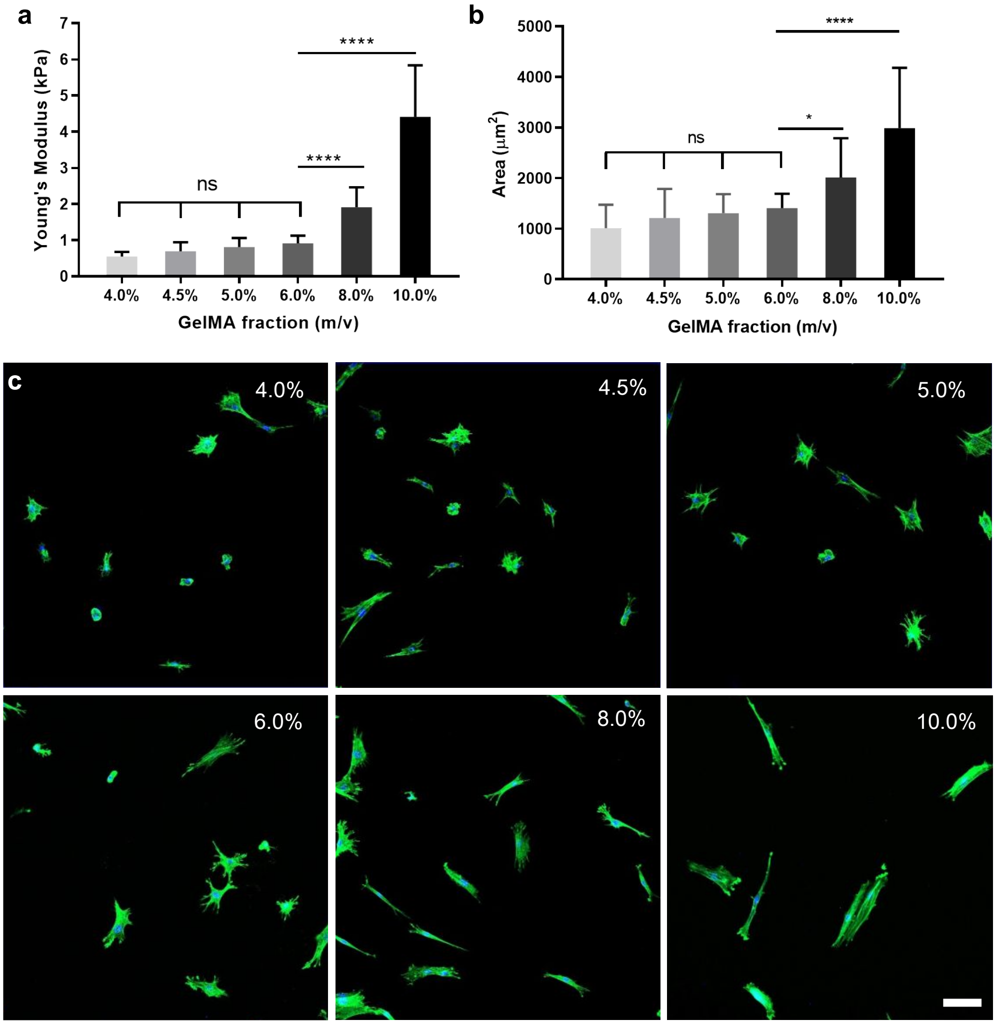

Figure S6. hMSCs adhesion on flat GelMA hydrogels with different fractions. (a) The Young's modulus of flat hydrogels fabricated with 4.0%, 4.5% 5.0%, 6.0%, 8.0% and 10% GelMA solution; (b-c) Cellular spreading area and fluorescent images of hMSCs after 12 hours incubation; the scale bar indicates 100 μm, green indicates cytoskeleton and blue indicates nucleus; N=20-30.

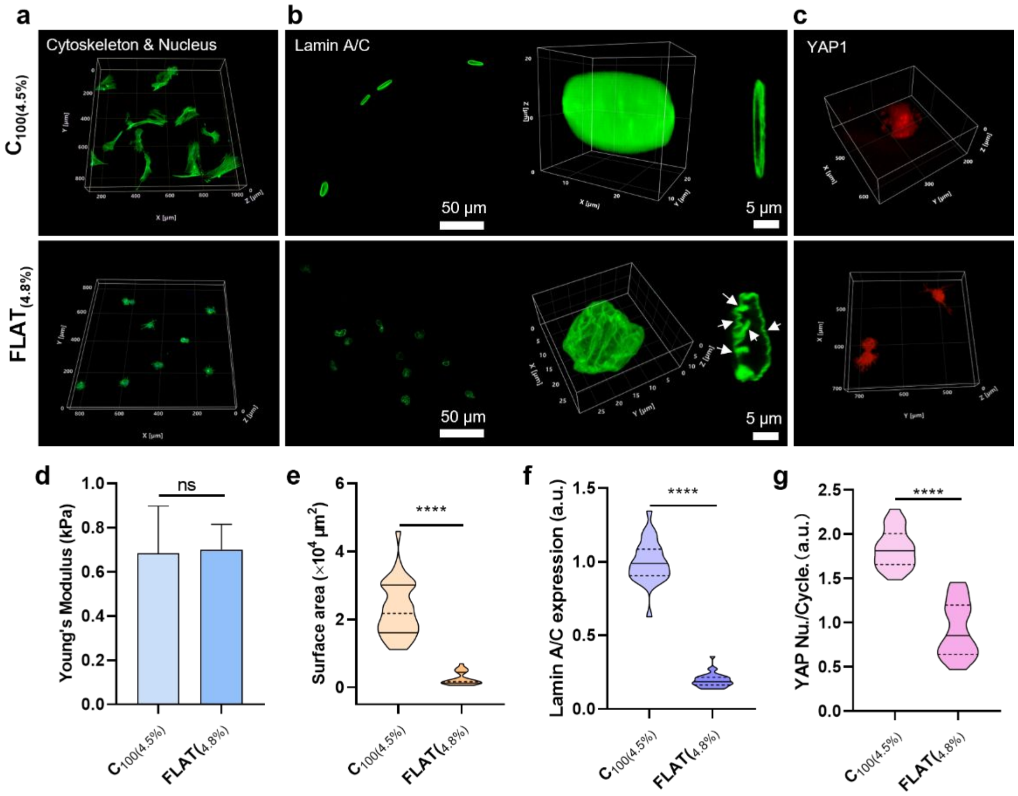

Figure S7. Cell mechanoresponse to the curvature features on ultrasoft substrates with similar young's modulus. Immunofluorescence staining of (a) F-actin (green), nuclei (blue), (b) Lamin A/C (green) and (c) anti-YAP (red) of hMSCs on 2D hydrogels with different curvatures. (d) The young's modulus of C_{100} and Flat hydrogels shows a similar value of ~690 Pa. (e-f) Quantification of cell surface area, Lamin A/C expression, and YAP nuclear/cytoplasmic ratio of hMSCs on soft hydrogel with varied curvatures. (N = 25–40, three technical replicates). $^{**}P < 0.01$, $^{***}P < 0.001$, unpaired t-test.

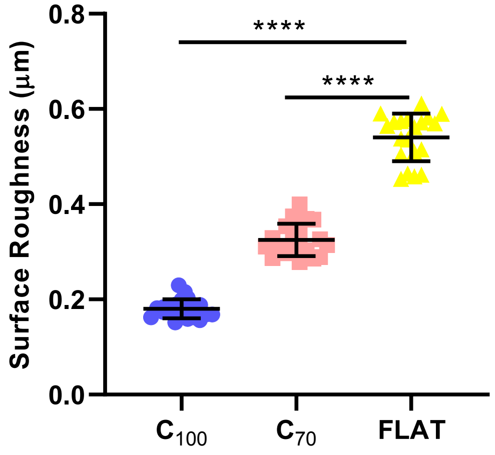

Figure S8. Surface curvatures regulate the morphology of cell nucleus. Quantification of nuclear surface roughness when cells adhered to different hydrogel surfaces. N = 20-30. Data represent mean ± SD. **P* < 0.05, ***P* < 0.01, ****P* < 0.001, one-way ANOVA.

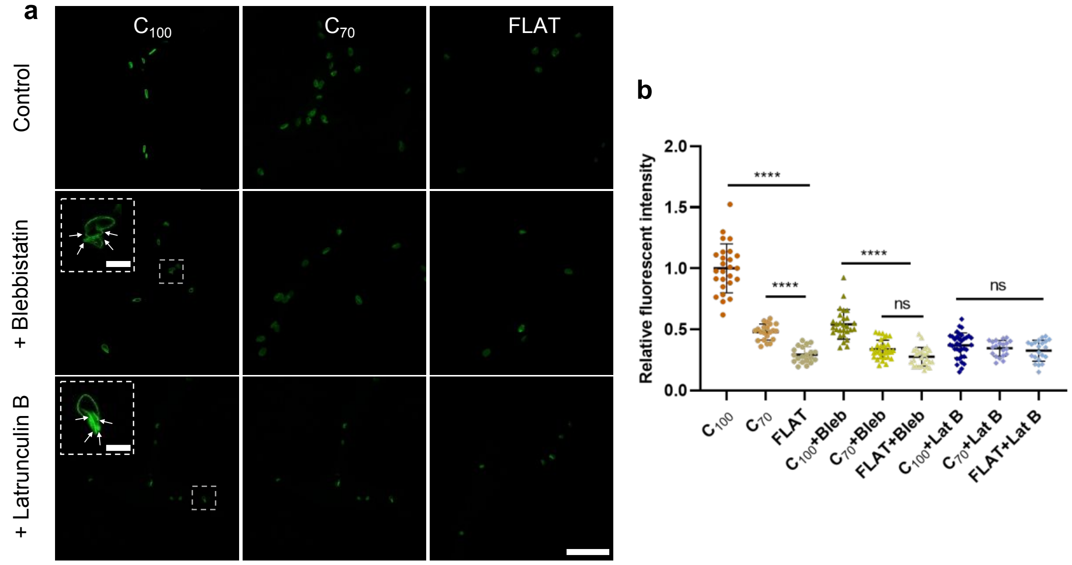

Figure S9. Expression of Lamin A/C when hMSCs were treated with 20 μM Blebbistatin and 0.2 μM Latrunculin B adhering to C_{100}, C_{70} and FLAT hydrogels. (a) The fluorescent images and (b) the quantification of Lamin A/C; the insets show the wrinkle generation on the nucleus after Blebbistatin and Latrunculin B treatment. The scale bars indicate 100 μm and 10 μm (inset), respectively; N=20-30.

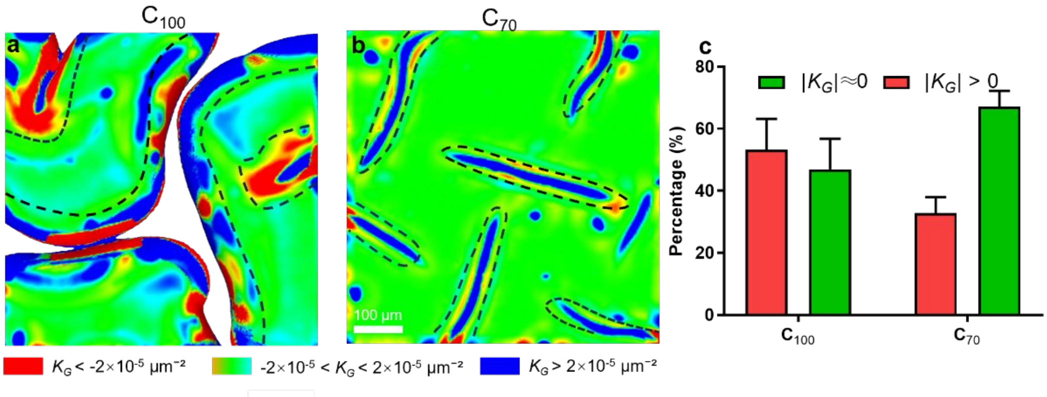

Figure S10. Estimating the distribution of quasi-uniaxial curvatures and multiaxial curvatures through calculation of surface Gaussian curvature (K_G). (a-b) Distributions of two defined curvatures on C_{100} and C_{70} surfaces respectively. Red and blue: multiaxial curvatures; intermediate colors: quasi-uniaxial curvatures. (c) The proportion of the two defined curvatures (quasi-uniaxial and multiaxial) on soft hydrogel after SICE treatment. We defined quasi-uniaxial areas as those with Gaussian curvature near zero ($-0.00002<K_G<0.00002$ μm^{-2}, labeled in green), and multiaxial areas as those where K_G exceeded this threshold (labeled in red/blue for positive/negative K_G, respectively). The black dashed lines show the area considered for quantitative statistics. The C_{100} and C_{70} samples correspond approximately to wrinkled regions with widths of 100 and 30 micrometers respectively, which are their corresponding wrinkle widths.

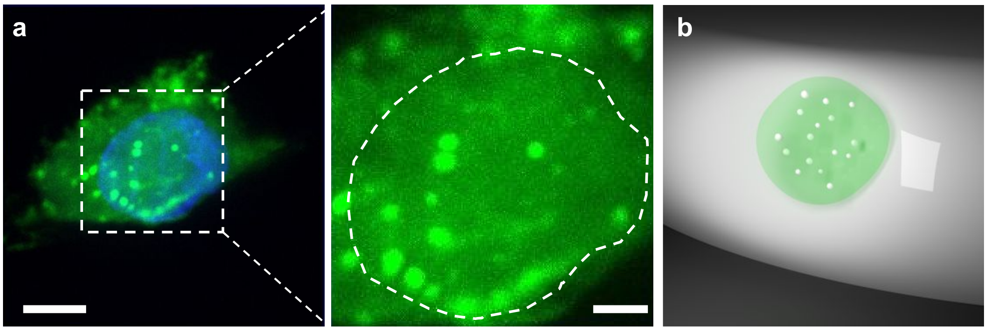

Figure S11. Actin of hMSCs treated 0.2 μM Latrunculin B. (a) The fluorescent images and (b) the schematic illustration of expressed actin indicate Latrunculin B treatment

inhibits actin fiber formation. The scale bar indicates 5 μm and 2 μm (zoom), respectively.

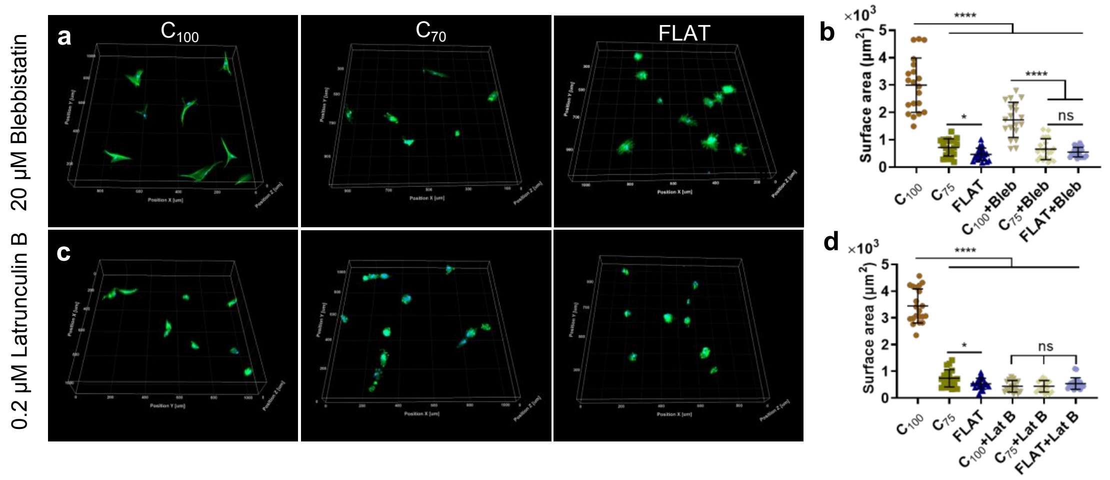

Figure S12. Adhesion of hMSCs treated with 20 μM Blebbistatin and 0.2 μM Latrunculin B. (a-b) The fluorescent images and surface area of hMSCs treated with 20 μM Blebbistatin; (c-d) The fluorescent images and surface area of hMSCs treated with 0.2 μM Latrunculin B; N=20-30.

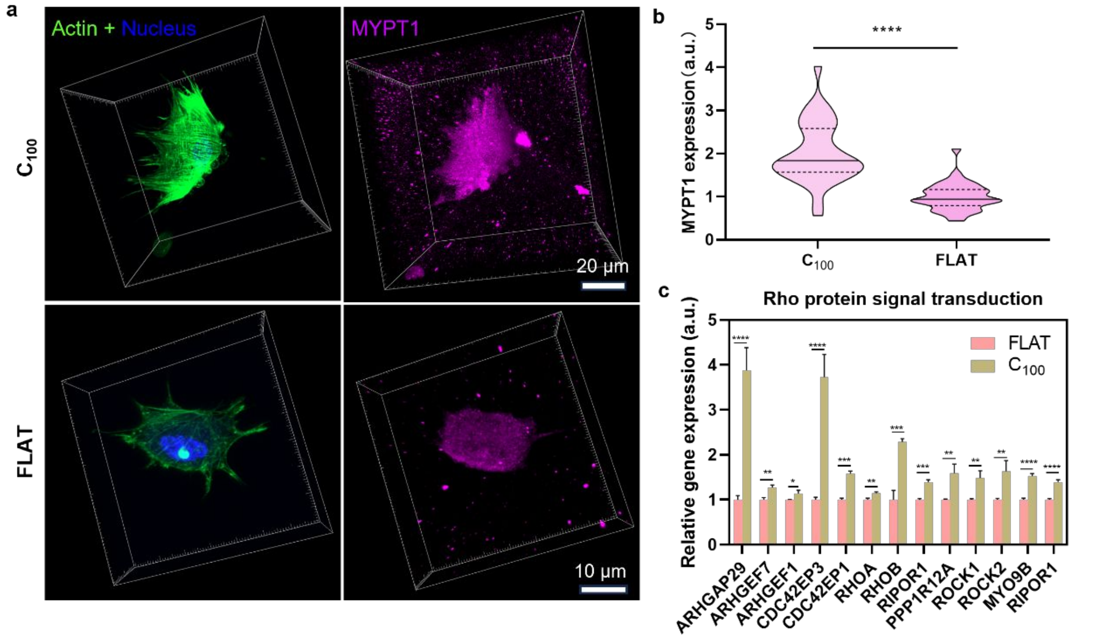

Figure S13. Multiaxial curvature activates the Rho/ROCK signaling pathway in

hMSCs. a) Representative immunofluorescence images of hMSCs cultured on FLAT and high-curvature (C_{100}) ultrasoft hydrogels for 24 hours, stained for F-actin (green), nuclei (blue), and myosin phosphatase targeting subunit 1 (MYPT1, magenta). b) Quantitative analysis of MYPT1 fluorescence intensity per cell, confirming significantly higher expression on C_{100} substrates compared to FLAT controls (**P < 0.01, unpaired t-test; N ≥ 30 cells from three independent experiments). Data are presented as mean ± SD. c) mRNA expression levels of key genes associated with Rho/ROCK signaling, derived from bulk RNA-seq data of hMSCs cultured on C_{100} *vs* FLAT substrates. Genes include Rho GTPases (RHOA, RHOB, RHOG), Rho effectors (ROCK1, ROCK2), regulatory proteins (ARHGAP29, CDC42EP3, MYO9B, RIPOR1), and the gene encoding MYPT1 (PPP1R12A). *$p < 0.05$, **$p < 0.01$, ***$p < 0.001$.

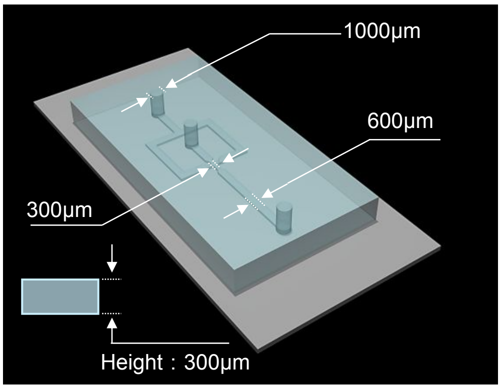

Figure S14. Schematic illustration of the designed microfluidic chip for microgel fabrication.

Table S2. Varying flow rate for the generation of microgels with different diameters.

	**100 μm**	**200 μm**	**500 μm**
$V_{Paraffin}$ (μL/min)	350	120	20
V_{GelMA} (μL/min)	20	20	25

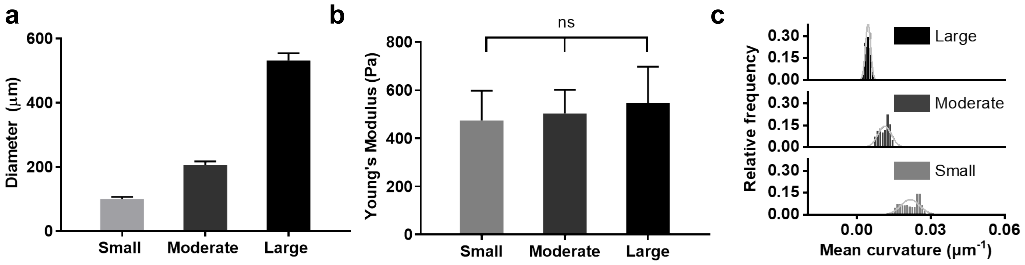

Figure S15. Characterization of fabricated smooth microgels. (a-c) The diameter, Young's modulus and mean curvature of fabricated smooth microgels.

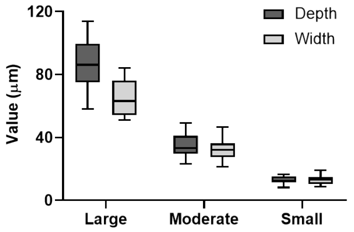

Figure S16. The width and depth of the curvature structures on curved microgels.

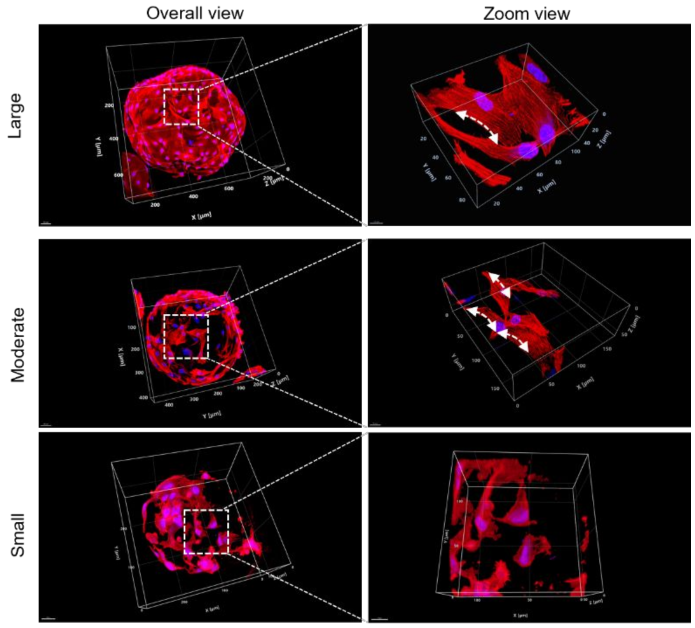

Figure 17. The fluorescent images of hMSCs attached to the curved microgels with different sizes. Cells were cultured on the curved microgels for 24 h. The white arrows

indicate cells adhering to one side of groove-like structures with bended morphology aligned with the substrate's curvature. Red: cytoskeleton; blue: nucleus.

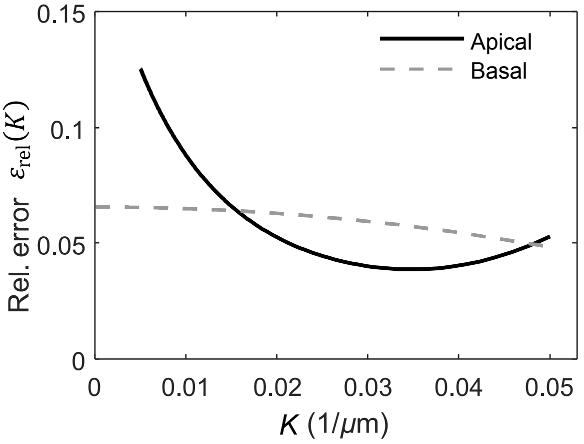

Figure S18. Normalized relative L_2-based fitting error between the prescribed arc-like stress-fiber (SF) profile and the Euler–Bernoulli beam approximation as a function of curvature $K(1/\mu\text{m})$. The solid black curve corresponds to the apical SF layer, and the dashed gray curve corresponds to the basal SF layer. The relative error is defined as $\varepsilon_{\rm rel}$ (K).

References

1. S. Chen, Y. Zhou, Y. Chen, J. Gu, fastp: an ultra-fast all-in-one FASTQ preprocessor. *Bioinformatics*. **34**, i884–i890 (2018).
2. D. Kim, J. M. Paggi, C. Park, C. Bennett, S. L. Salzberg, Graph-based genome alignment and genotyping with HISAT2 and HISAT-genotype. *Nat. Biotechnol.* **37**, 907–915 (2019).
3. Y. Liao, G. K. Smyth, W. Shi, FeatureCounts: An efficient general purpose program for assigning sequence reads to genomic features. *Bioinformatics*. **30**, 923–930 (2014).
4. Z. Cai, D. H. Kwak, D. Punihaole, Z. Hong, S. S. Velankar, X. Liu, S. A. Asher, A Photonic Crystal Protein Hydrogel Sensor for Candida albicans. *Angew. Chemie - Int. Ed.* **54**, 13036–13040 (2015).
5. M. Werner, N. A. Kurniawan, G. Korus, C. V. C. Bouten, A. Petersen, Mesoscale substrate curvature overrules nanoscale contact guidance to direct bone marrow stromal cell migration. *J. R. Soc. Interface*. **15**, 20180162 (2018).

6. A. Elosegui-Artola, R. Oria, Y. Chen, A. Kosmalska, C. Pérez-González, N. Castro, C. Zhu, X. Trepat, P. Roca-Cusachs, Mechanical regulation of a molecular clutch defines force transmission and transduction in response to matrix rigidity. *Nat. Cell Biol.* **18**, 540–548 (2016).
